# Human NORs, comprising rDNA arrays and functionally conserved distal elements, are located within dynamic chromosomal regions

**DOI:** 10.1101/gad.331892.119

**Published:** 2019-12-01

**Authors:** Marjolein van Sluis, Michael Ó Gailín, Joseph G.W. McCarter, Hazel Mangan, Alice Grob, Brian McStay

**Affiliations:** Centre for Chromosome Biology, School of Natural Sciences, National University of Ireland Galway, Galway, Ireland

**Keywords:** nucleolar organizer regions (NORs), acrocentric chromosomes, ribosomal DNA, nucleolus, recombination

## Abstract

Human nucleolar organizer regions (NORs), containing ribosomal gene (rDNA) arrays, are located on the p-arms of acrocentric chromosomes (HSA13–15, 21, and 22). Absence of these p-arms from genome references has hampered research on nucleolar formation. Previously, we assembled a distal junction (DJ) DNA sequence contig that abuts rDNA arrays on their telomeric side, revealing that it is shared among the acrocentrics and impacts nucleolar organization. To facilitate inclusion into genome references, we describe sequencing the DJ from all acrocentrics, including three versions of HSA21, ∼3 Mb of novel sequence. This was achieved by exploiting monochromosomal somatic cell hybrids containing single human acrocentric chromosomes with NORs that retain functional potential. Analyses revealed remarkable DJ sequence and functional conservation among human acrocentrics. Exploring chimpanzee acrocentrics, we show that “DJ-like” sequences and abutting rDNA arrays are inverted as a unit in comparison to humans. Thus, rDNA arrays and linked DJs represent a conserved functional locus. We provide direct evidence for exchanges between heterologous human acrocentric p-arms, and uncover extensive structural variation between chromosomes and among individuals. These findings lead us to revaluate the molecular definition of NORs, identify novel genomic structural variation, and provide a rationale for the distinctive chromosomal organization of NORs.

## Abstract (precis)

In this study, van Sluis et al. investigated the role of chromosomal context in nuclear organizer regions (NORs)/ribosomal gene (rDNA) in nucleolar formation and function. The analyses combined sequence capture and long-read sequencing to characterize the regions distal to rDNA arrays (DJ) on isolated acrocentric chromosomes, and their findings provide direct evidence for exchanges between heterologous human acrocentric p-arms, and uncover extensive structural variation between chromosomes and among individuals.

Nucleolar organizer regions (NORs), comprising ribosomal gene (rDNA) repeat arrays, are the most heavily expressed regions of eukaryotic genomes. When transcribed by a dedicated RNA polymerase I (Pol I) transcription machinery, NORs seed formation of nucleoli, the sites of ribosome biogenesis and the largest substructure in eukaryotic nuclei (Raška et al. 2006; Hernandez-Verdun 2011; Mangan et al. 2017). Despite nucleoli and ribosome biogenesis being at the core of cellular life, NORs are underexplored from a genomic perspective (McStay 2016).

In humans, rDNA arrays ranging from 50 kb (∼1 rDNA repeat) to 6 Mb (∼140 repeats) are located on the p-arms of the five acrocentric chromosomes, HSA13–15, HSA21, and HSA22 (Fig. 1A; Henderson et al. 1972; Stults et al. 2008, 2009). Acrocentric chromosome p-arms are among the more prominent regions missing from human genome drafts (McStay 2016). While the sequence of individual rDNA repeats has been known for some time (Gonzalez and Sylvester 1995), only recently are efforts beginning toward sequencing entire rDNA arrays (Kim et al. 2018). We reasoned that sequences either on the proximal (centromeric) or distal (telomeric) sides of rDNA arrays could play roles in NOR activation, nucleolar formation, or ensuring genomic stability of rDNA arrays. Building on earlier reports of sequences distal and proximal to the rDNA array on HSA21 and HSA22, respectively (Worton et al. 1988; Sakai et al. 1995; Gonzalez and Sylvester 1997), we identified 207 kb of sequence immediately proximal and 379 kb distal to rDNA arrays (Floutsakou et al. 2013). Consensus proximal junction (PJ) and distal junction (DJ) contigs were assembled using sequenced bacterial artificial chromosomes (BACs) that were either unplaced or misplaced on the human genome. Hybridization of these BACs to normal human metaphase spreads revealed that these sequences are shared between all five acrocentric chromosomes. PJ and DJ sequences exhibit contrasting genomic characteristics. PJ sequences are almost entirely segmentally duplicated, similar to the regions bordering centromeres, and are unlikely to contain any specific elements that would regulate activity of the linked NOR. In contrast, DJ sequences are predominantly confined to the acrocentric short arms and are dominated by a very large inverted repeat of >100 kb (Fig. 1B). We identified a large (∼40 kb) block of 48-bp satellite repeats, CERs, at the distal end of the DJ and showed that CER blocks are found distal to the rDNA on all acrocentric chromosomes, with additional pericentromeric blocks on HSA14 and HSA22.

**Figure 1.**
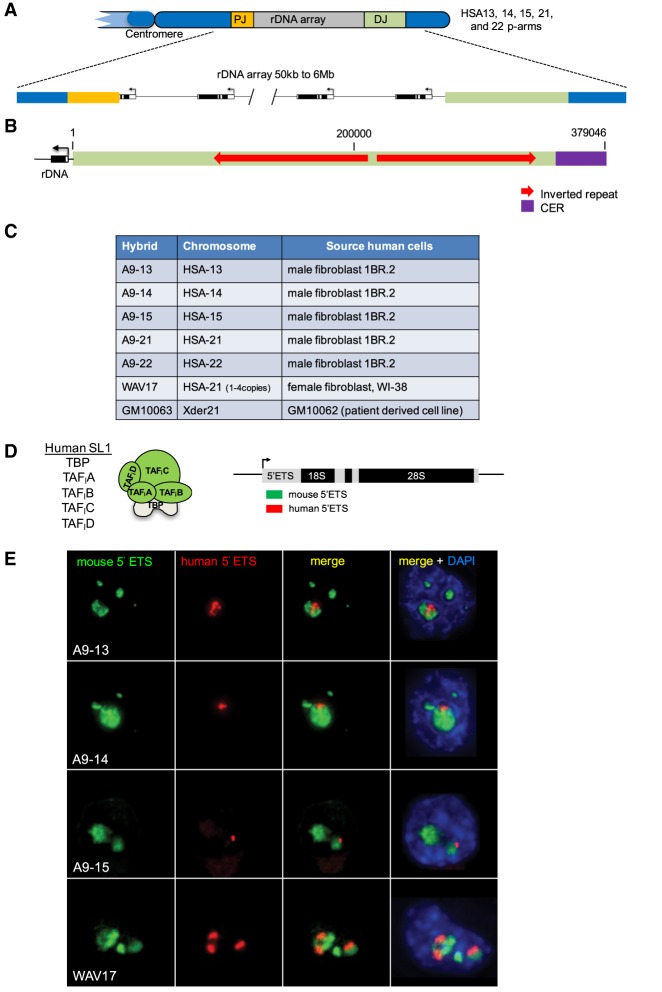
NORs within monochromosomal hybrids retain functional potential. (*A*) Schematic diagram of the rDNA arrays on the p-arms of the five acrocentric human chromosomes. (PJ) Proximal junction, (DJ) distal junction. (*B*) Schematic diagram of the original 379-kb DJ contig showing the location of large inverted repeats and CER satellite. (*C*) List of monochromosomal hybrids, showing the human acrocentric chromosome they contain and the human cell line source of this chromosome. (*D*) A schematic representation of human SL1 and the mouse and human 5′ ETS RNA FISH probes. (*E*) RNA FISH experiments performed on hybrid cells transfected with expression plasmids encoding human TAF_I_ A–D.

By including the DJ into a customized human genome reference and remapping ENCODE data sets, we established the chromatin and transcriptional profile across this DJ consensus in a variety of human cell types (Floutsakou et al. 2013). The DJ has a complex chromatin landscape that is largely conserved among cell types. Each arm of the inverted repeat contains chromatin signatures for promoters and actively transcribed gene bodies in all cell types analyzed. Spliced and polyadenylated RNA polymerase II transcripts, apparent long noncoding RNAs (lncRNAs), corresponding to these DJ sequences were readily identified. A functional role for DJ sequences was further inferred by their localization within perinucleolar heterochromatin, where they appear to anchor linked rDNA arrays that extend into the nucleolar interior (Floutsakou et al. 2013; van Sluis and McStay 2015).

Future research into the role of the DJ and further distal regions in regulation of NORs requires that we obtain sequences from all five acrocentric chromosomes. This would additionally facilitate their inclusion in an updated genome reference, making them visible to the larger research community. Here, we utilize a panel of monochromosomal somatic cell hybrids in which individual human acrocentric chromosomes are held within a mouse A9-cell. We demonstrate that the functionality of these NORs has been retained by reactivating them in this context. We combine sequence capture and single-molecule long-read sequencing to determine both the DJ and further distal sequences from seven chromosomes, including three different versions of HSA21. We reveal a remarkable degree of sequence similarity over the ∼300 kb immediately distal to rDNA arrays. Functional conservation is demonstrated by the fact that the DJ present on all acrocentric chromosomes yields transcripts that when depleted result in nucleolar stress. Conservation is further confirmed by identification of “DJ-like” sequences and transcripts in chimpanzees (*Pan troglodytes*). Sequence analysis also provides evidence for exchanges between heterologous human acrocentric chromosomes and reveals a high level of inter-individual structural variation in further distal regions of these chromosomes.

## Results

### Human NORs in monochromosomal somatic cell hybrid cells retain functionality

The similarity of NOR distal regions between the acrocentrics necessitates that we determine sequences from individual isolated chromosomes, and their intrinsic repeat sequence composition requires that we employ long-read sequencing technologies. To isolate individual human chromosomes, we exploited a panel of monochromosomal somatic cell hybrids A9-13, A9-14, A9-15, A9-21, and A9-22 (Cuthbert et al. 1995; Sullivan et al. 2001). These are mouse A9 cells containing a single human acrocentric chromosome from the human male fibroblast cell line 1BR.2 (Fig. 1C). We also included WAV17 (Coriell; GM08854), A9 cells containing one to five copies of an identical HSA21 from the normal diploid human female fibroblast line WI-38. Additionally, GM10063 contains a patient-derived der(X)t(X;21) product of a reciprocal translocation between the dystrophin gene on HSAX and the rDNA array on HSA21 (Worton et al. 1984). GM10063 contains the three most distal rDNA repeats and NOR distal sequences from HSA21. Consequently, our hybrid panel contains three versions of HSA21 distal sequences (A9-21, WAV17, and GM10063), allowing us to explore variation at the DNA sequence level within an individual acrocentric chromosome. The additional advantage gained from using GM10063 is that we could be certain that the sequences obtained are distal to rDNA. The identity of the human chromosome in each of the hybrids was confirmed by PCR amplification of sequence-tagged site (STS) markers specific for each chromosome (Supplemental Fig. S1A). The presence of DJ sequences was demonstrated by probing metaphase spreads from hybrid lines with the DJ BAC CT476834 (Supplemental Fig. S1B).

As our initial motivation for sequencing NOR distal regions was largely related to studying their role in nucleolar formation and function, we thought it important to demonstrate that the acrocentric p-arm sequences held within these hybrids are intact and retain the potential to participate in nucleolar formation. Previously, we had demonstrated that human NORs held in such hybrid lines are transcriptionally silent (Sullivan et al. 2001). This is a consequence of species specificity in the RNA Pol I transcription machinery. Specificity resides in the SL1 transcription factor (Heix and Grummt 1995) that is comprised of TBP and RNA Pol I-specific TBP-associated factors, TAF_I_A–D (Zomerdijk et al. 1994; Gorski et al. 2007). Despite their transcriptional silence, we observed that human NORs in hybrid lines are both nucleolar-associated and loaded with upstream binding factor, UBF, the mitotic bookmark of active NORs (Sullivan et al. 2001; Grob et al. 2014). We hypothesized that these human NORs are in a poised state and can be reactivated by supplying human SL1 in *trans*. To test this, hybrid lines were transfected with plasmids expressing the four human RNA Pol II-specific TAFs, TAF_I_A–D (Fig. 1D). RNA FISH performed on transfected hybrid lines using both mouse- and human-specific 5′ ETS probes revealed that human NORs are poised for reactivation and that functionality of NORs within hybrid lines is retained (Fig. 1D,E). Quantitation of fluorescence signals in transfected cells indicated that levels of transcription from the reactivated human NOR are comparable to mouse NORs (Supplemental Fig. S2).

### DNA sequencing of rDNA distal regions from individual human acrocentric chromosomes

Sequencing of the human acrocentric NOR distal regions within each of these hybrids was achieved by combining sequence capture with PacBio Single Molecule, Real-Time (SMRT) sequencing. The work flow is illustrated in Supplemental Figure S3. Precapture libraries were prepared using size fractionated (typically 4–6 kb) genomic DNA from each hybrid line. A capture oligo probe set was designed using the original DJ contig sequence (Floutsakou et al. 2013), constructed from a series of sequenced BAC clones mostly derived from chromosome 21 (Supplemental Fig. S4A). Nucleotides 1 to 297192 of our original DJ contig were assembled using sequenced BAC clones (GenBank: CT476837, CT476834, and CU633906) from a human genomic library, CHORI-507-HSA21 (Lyle et al. 2007), prepared from WAV17. The remainder of the contig, including the terminal CER satellite array, was derived from a single sequenced BAC clone RP11-272E10 (GenBank: AC011841), from RPC-11 Human Male BAC library, incorrectly assigned to HSA17.

Circular consensus sequencing (CCS) of postcapture libraries was performed. Extracted reads of insert (ROIs) from CCS (>3 passes per insert) have sufficient accuracy (∼98%) to build sequence contigs de novo through repetitive DNA, including inverted repeats and blocks of CER satellites. In most cases, data from a single SMRT cell provided sufficient depth of coverage (200–500×). A summary of the sequencing statistics is presented in Supplemental Table S1, and read coverage maps are presented in Supplemental Figures S5A and S6. The read coverage maps reveal a number of recurring small gaps (50–200 nt) due to short stretches of pure GC- or AT-rich DNA (Supplemental Fig. S5A). These gaps were filled by direct sequencing of targeted PCR products.

The validity of this sequencing strategy is demonstrated by comparison of the DJ contig we obtained from HSA21 in the WAV17 hybrid (MG910340) with fully sequenced BAC clones from the same hybrid. BAC clones CH507-535F5 (Genbank: CT476834) and CH507-145C22 (Genbank: CU633906) each show only ∼25 nucleotide differences over their respective 183 and 166 kb (Supplemental Fig. S5B).

**Figure 2.**
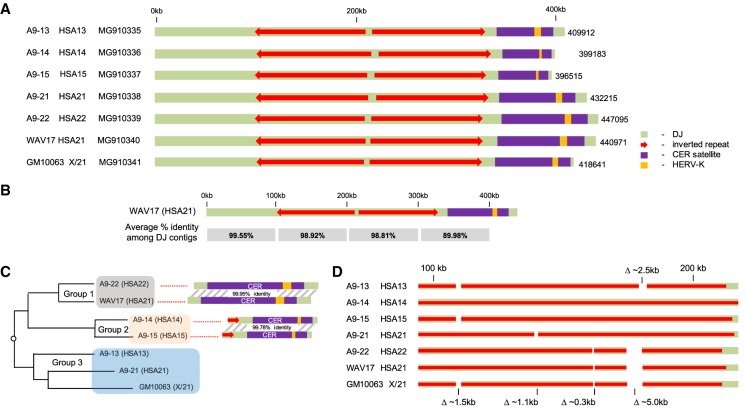
Sequencing of DJ regions from individual human acrocentric chromosomes. (*A*) Schematic representations of DJ contigs. Hybrid and chromosomal identities are shown on the *left*, together with GenBank accession numbers. (*B*) The average percentage identity of 100-kb blocks, among all seven DJ contigs is shown *below* the WAV17 (HSA21) contig. (*C*) Alignment of DJ contigs demonstrating that they can be clustered into three groups, based on the sequence of their distal ends. The percentage identity of the most distal sequences within group 1 and 2 members is shown schematically on the *right*. (*D*) A schematic representation of indel distribution on the left arm inverted repeat. Hybrid and chromosomal identities are shown on the *left* (see Supplemental Fig. S7B for sequence alignments at break points).

Graphic representations of the DJ contigs we have constructed from all seven hybrid lines are shown in Figure 2A. All hybrids have inverted repeats of >100 kb, large blocks of CER satellites with embedded integrations of human endogenous retrovirus type K (HERV-K)-related sequences. In all, this provides 2.945 Mb of novel sequence (Genbank: MG910335–MG910341). Sequences from A9-13 to A9-22 form a major part of a recent patch update to the human genome reference, GRCh38.p13. Captured fragments were up to 6 kb in size and extended into sequences not targeted by the capture oligo probes. Thus, our contigs contain on average 4 kb of rDNA on one end and novel non-CER satellite sequence at the other. We were also able to sequence across most HERV-K integrations. However, the two large (∼9 kb) HERV-K integrations, with high sequence homology to each other and HERV-K111 (Genbank: GU476554) on A9-22 and WAV17, were completed by sequencing targeted PCR products.

The availability of linked rDNA sequence has allowed us to demonstrate that the sequence at the break point between rDNA and the DJ is identical at the nucleotide level in all the chromosomes analyzed and lies ∼4 kb upstream of the pre-rRNA transcriptional start site (Supplemental Fig. S7A). In contrast, the position of the PJ-rDNA breakpoint is variable between the acrocentrics (Floutsakou et al. 2013).

By using a sliding window of 100 kb across the WAV17 DJ contig, BLAST was used to calculate the average percentage identity between all DJ contigs across their length (Fig. 2B). Within the first 300 kb, the percentage identity between all seven contigs averages at ∼99%. Over the next 100 kb, including CER satellites, the average percentage identity drops to below 90%. However, within this region we observe that the A9-22 (HSA22) DJ contig has 99.96% sequence identity with the WAV17 (HSA21) contig. This suggested that DJ contigs could be organized into groups based on their sequence composition.

Multiple sequence alignments of all DJ contigs were performed using MAFFT (Multiple Alignment using Fast Fourier Transform) (Katoh et al. 2002). The overall similarities of contigs are displayed by a relationship tree and can be classified into three groups (Fig. 2C). As the termini of DJ contigs show the most variability, classification of DJ contigs is driven primarily by sequences at their distal ends. The final 112 kb of group 1 contigs, A9-22 (HSA22) and WAV17 (HSA21), share 99.95% sequence identity (53 nucleotide differences). In group 2 contigs from A9-14 (HSA14) and A9-15 (HSA15), their final 75 kb share 99.78% sequence identity. Similarly, group 3 contigs, A9-13 (HSA13), A9-21 (HSA21), and GM10063 (Xder21), have almost identical sequences at their distal ends. We have observed three different HERV-K integration events within CER satellite blocks. Interestingly, within each group, precisely identical integrations are observed.

Alignments reveal the presence of indels, ranging from tens of nucleotides up to 5 kb, within the conserved first 300 kb of DJ contigs. Interestingly, the majority of the larger indels reside within the left arm inverted repeat. These are represented graphically in Figure 2D. A striking feature of these indels is their distribution among the sequenced chromosomes. A 1.5-kb deletion, positioned at 110 kb on the WAV17 contig, is present on DJ contigs from A9-13 (HSA13), A9-15 (HSA15), and GM10063 (Xder21). Sequence alignments reveal that the deletion break points are identical on all three contigs despite the fact that they are derived from three different acrocentrics (Supplemental Fig. S7B). Likewise, deletions of 0.3 and 5 kb, positioned at 161 and 172 kb, respectively, on the WAV17 contig, are present in contigs from A9-22 (HSA22), WAV17(HSA22), and GM10063(Xder21). Sequence alignments reveal that deletion break points are identical on all three chromosomes (Supplemental Fig S7B). The sequence identity between chromosomes, indel distribution, and identical HERV-K integrations (Fig. 2C,D; Supplemental Fig. S7B) confirm that exchanges between heterologous acrocentric chromosomes have occurred within the DJ region. For example, exchanges appear to have occurred in the 50-kb interval between Δ1.5 and Δ0.3 kb. However, no exchanges are observed in the 11-kb interval between Δ0.3 and Δ5.0 kb.

### Functional conservation of DJ transcripts

**Figure 3.**
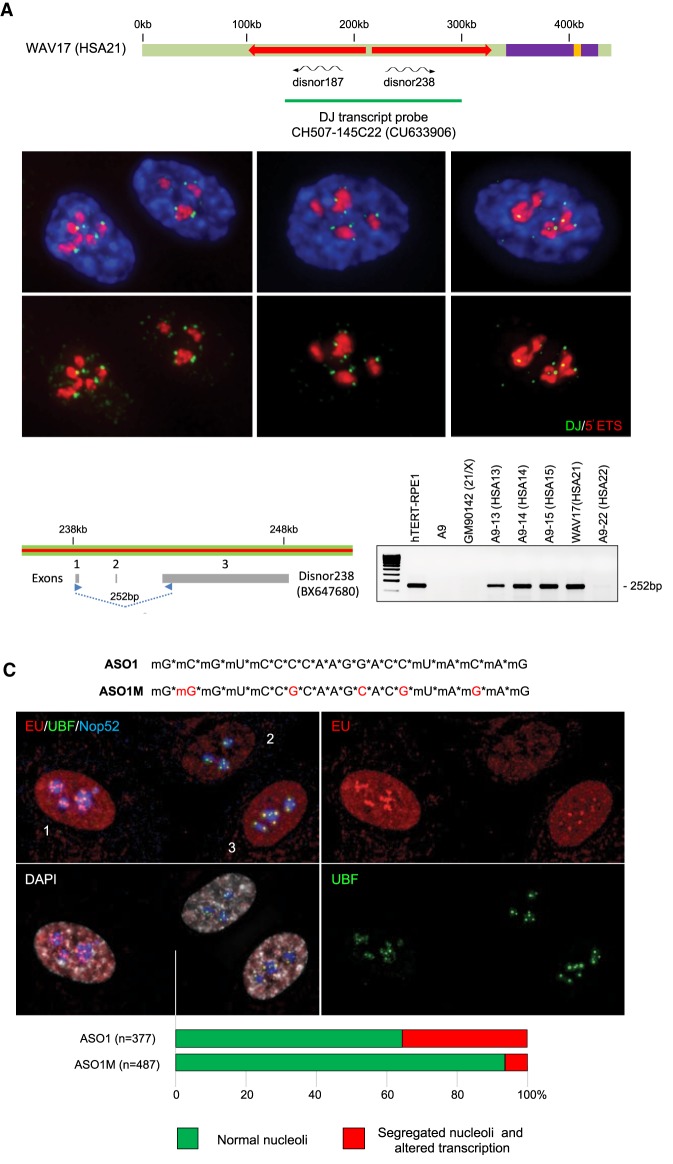
DJ transcripts, arising from each acrocentric chromosome are essential for nucleolar function. (*A*) A schematic of the DJ showing location of left arm and right arm transcripts, disnor 187 and disnor 238, respectively. *Below*, results from RNA FISH performed on hTert-RPE1 cells with BAC clone CH507-145C22 (CU633906) to detect DJ transcripts, labeled in green, together with a human 5′ ETS probe in red. (*B*) Detection of disnor238 in monochromosomal somatic cell hybrid lines by RT-PCR with primers from exon 1 and 3. A9 and GM09142 (DJ-negative) cells served as negative controls and hTert-RPE1 as a positive control. (*C*) Twenty-nucleotide chimeric antisense oligonucleotides (ASO1 and 1M) comprise 5-nt 2-O-methoxyribonucleotide segments at both termini and a deoxynucleotide segment containing 10 central nucleotides. Inter-nucleotide linkages were phosphorothioate (*). hTert-RPE1 cells were transfected with ASO1 and 1M and analyzed 24 h later by immunofluorescent staining with antibodies against UBF and NOP52, combined with EU incorporation to monitor ongoing transcription. Effective depletion of DJ transcripts is demonstrated in Supplemental Figure S8. Representative cells, indicated as 1–3, are described in the main text. Quantitation of the experiment is shown *below*. A further two independent experiments provided essentially the same result (data not shown).

Mapping of ENCODE data sets onto our original DJ consensus sequence predicted the existence of spliced and polyadenylated transcripts, presumed lncRNAs, arising from each inverted repeat arm (Floutsakou et al. 2013). The left and right arm transcripts were termed disnor 187 and disnor 238 due to the position, in kilobases, of their transcription start sites on the original consensus DJ. Disnor 187 and 238 are virtually identical to sequenced cDNA clones (GenBank: AK026938 and BX647690, respectively). These transcripts could be readily detected by reverse transcriptase PCR (RT-PCR), but at the time we could not confirm that all acrocentric chromosomes were capable of producing such transcripts. The sequence data described here indicate that all acrocentrics could in principle produce left and right arm transcripts. To test this, we performed RNA FISH on hTERT-RPE1 cells with a 5′ ETS probe visualising pre-rRNA together with HSA21 BAC clone CH507-145C22 (GenBank: CU633906). This BAC probe spans both left- and right-arm DJ transcripts (Fig. 3A). In these experiments, we observed up to 10 DJ transcript signals of varying intensity associated with the nucleolar periphery. The two most reasonable interpretations are that we observed either nascent transcripts or released transcripts that remain close to their site of synthesis, as has been observed for other lncRNAs (Bassett et al. 2014; Kopp and Mendell 2018).

As RNA polymerase II is not known to be species-specific, we also investigated if DJ transcripts were expressed in the monochromosomal hybrid lines. Using primers designed against exons 1 and 3 of the right-arm transcript (disnor238, BX647680), we could readily detect spliced transcripts from HSA13, 14, 15, and 21. HSA22 gave a weak but detectable signal (Fig. 3B). Confirming that these transcripts are derived from the DJ, we demonstrate that they are absent in hybrid line GM09142. This hybrid contains a 21derX translocation comprising all of HSA21, except for NOR distal sequences. These combined results confirm that each acrocentric chromosome is capable of producing DJ transcripts.

Sequence identity among the DJ contigs allowed us to design a single antisense oligonucleotide that could simultaneously target for depletion in both left- and right-arm transcripts from all five acrocentric chromosomes. Accordingly, we designed chemically modified chimeric antisense oligonucleotides (ASOs) as described previously (Ideue et al. 2009). ASO1 targets exon 1 of both left- and right-arm transcripts from all acrocentrics, while ASO1M has five mismatches incorporated and served as a negative control (Fig. 3C). After introduction of ASOs into hTERT-RPE1 cells by electroporation, RT-PCR analysis using primers from exons 1 and 3 of the proposed right-arm transcript, disnor238, reveal that ASO1 effectively depleted this transcript (Supplemental Fig. S8). Similarly, primer pairs from exon 3 of this transcript and exon 4 of the proposed left-arm transcript, disnor187, confirm that both transcripts have been depleted across their length (Supplemental Fig. S8). Cell numbers were monitored for up to 5 d postelectroporation. We consistently observed an approximately twofold drop in cell numbers in ASO1- versus ASO1M-transfected cells (Supplemental Fig. S9).

Reduction in cell numbers may arise as a consequence of slow growth in all ASO1-transfected cells or cell cycle arrest in a proportion of cells coupled with outgrowth of unaffected cells. To address this, we determined cell cycle profiles by utilizing the FUCCI system to distinguish cells in G1 and G2 (Sakaue-Sawano et al. 2008) and EdU incorporation followed by click-chemistry to reveal cells in S-phase (Supplemental Fig S10). These experiments revealed that, at 48 h posttransfection, cell cycle profiles of ASO1 and ASO1M cells were indistinguishable. This suggested that, at early times posttransfection, a proportion of ASO1-treated cells were seriously compromised. Accordingly, we examined nucleolar organization and transcription by RNA Pol I at 24 h posttransfection. In a high proportion of ASO1-transfected cells (∼35%), we observe nucleolar segregation, a hallmark of nucleolar stress (Mangan et al. 2017). This was revealed by formation of UBF-positive nucleolar caps on the periphery of a NOP52-positive nucleolar core. Within these cells, RNA Pol I transcription, revealed by nucleolar-associated EU incorporation, was altered (Fig. 3C). In cells such as #2 in Figure 3C, nucleolar transcription is absent. In some cells with segregated nucleoli, typified by #3, RNA Pol I transcription is restricted to nucleolar caps. Importantly, cells with segregated nucleoli were far less common in transfections with ASO1M. Finally, in cells with segregated nucleoli, transcription by RNA Pol II also appears to be reduced (see #2 in Fig. 3C). At the moment, we are uncertain of the fate of these cells. As we see no evidence for apoptosis, we suspect that they exit the cell cycle. Nevertheless, we can now be certain that DJ transcripts play an essential role in nucleolar function.

### Identification of DJ-related sequences in chimpanzee

**Figure 4.**
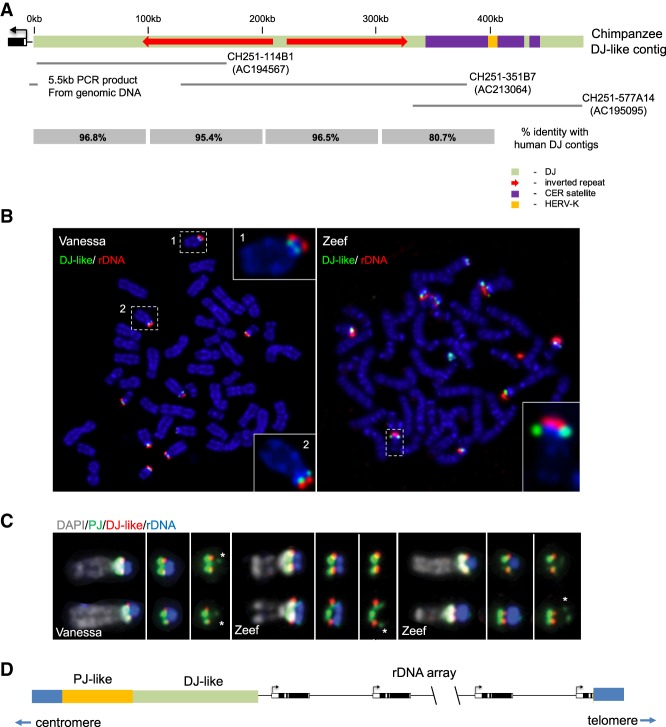
Identification of “DJ-like” sequences in the chimpanzee, *Pan troglodytes.* (*A*) A schematic representation of the chimpanzee “DJ-like” contig, illustrating sequence composition and positions of BAC clones and cloned PCR products used in its construction. The percentage identity, 100-kb blocks, with human DJ contig from WAV17 (HSA21) is shown *below*. (*B*) DAPI (blue)-stained metaphase spreads prepared from B-cells of two individual chimpanzees, Vanessa (female) and Zeef (male), probed with a mixture of “DJ-like” BAC clones CH251-114B1 and CH251-351B7 (green) and a human rDNA probe (red). *Insets* show enlarged acrocentric chromosomes. (*C*) Individual acrocentric chromosomes from Vanessa (female) and Zeef (male) metaphase spreads probed with “DJ-like” chimpanzee BAC clones (red), human PJ BAC clone bP-2154M18 (CR381535) (green), and rDNA (far red, pseudocolored here in blue). Chromosomes were DAPI-stained (pseudocolored in gray). Full metaphase spreads are presented in Supplemental Figure S11B. (*D*) A schematic illustrating the inverted orientation of rDNA and “DJ-like” sequences in chimpanzees.

The chimpanzee, *Pan troglodytes*, karyotype is identical to that of humans other than chromosomes 2A and 2B remaining unfused (Chimpanzee Sequencing and Analysis Consortium 2005). As in humans, NORs are present on chimpanzee chromosomes 13, 14, 15, 21, and 22. Overlapping BAC clones CH251-114B1 (AC194567), CH251-351B7 (AC213064), and CH251-577A14 (AC195095), incorrectly annotated as from chromosome 7, can be used to build a sequence contig that has high similarity to the human DJ (Fig. 4A). In order to confirm that these BACs represent sequences adjacent to chimpanzee NORs, metaphase spreads prepared from B-cells of two individual chimpanzees, Vanessa and Zeef, were probed with BACs AC194567 and AC213064 together with a human rDNA probe (Fig. 4B). Adjacent signals can be observed on the p-arms of the chimpanzee acrocentric chromosomes. Based on sequence alignments, a contig constructed from chimpanzee BAC sequences would be predicted to originate ∼5 kb from the rDNA junction. To confirm that the rDNA/DJ junction is similar to humans, PCR was performed with an rDNA primer combined with a primer near the start of the proposed BAC contig and using genomic DNA from two chimpanzee individuals, Vanessa and Walter. The design of the rDNA primer was facilitated by the availability of a reference sequence for the chimpanzee rDNA repeat (KX061886.1). Five cloned 5.5-kb PCR products from the two chimpanzees were sequenced (GenBank: MN646268-72) They confirmed linkage of the BAC contig to rDNA (Fig 4A). Minor sequence differences between the PCR clones are observed, confirming that they are from different chromosomes and not PCR duplicates. However, the sequence across the precise rDNA/DJ junction is identical in each case (Supplemental Fig. S11A). This proposed chimpanzee “DJ-like” contig has the major defining features of human DJ contigs, including inverted repeat arms and a CER satellite array. The CER block also contains an integrated HERV-K that has ∼98% sequence identity to the 9-kb HERV-K found in WAV17 and A9-22 human DJ contigs. As observed among human DJ sequences, there is high sequence conservation between human and chimpanzee over the first 300 kb of DJ sequence, ∼96% identity, dropping to ∼80% identity after 300 kb (Fig. 4A).

In humans, DJ sequences are clearly positioned on the distal/telomeric side of rDNA on acrocentric chromosomes (Floutsakou et al. 2013). In contrast, on all chimpanzee chromosome spreads (>100) we have analyzed to date, the equivalent sequences appear proximal to rDNA. To further explore this, chimpanzee chromosome spreads were additionally probed with BAC clone bP-2154M18 (GenBank: CR381535) representing the human PJ (Floutsakou et al. 2013). In a centromeric to telomeric direction, hybridization signals are observed in the following order PJ-DJ-rDNA (Fig. 4C; Supplemental Fig. S8B). On a number of chimpanzee chromosomes, we also observe a weak PJ hybridization signal on the distal side of the rDNA (indicated by an asterisk in Fig. 4C). This extra signal is due to segmental duplication of PJ sequences, as previously observed on human chromosomes (Floutsakou et al. 2013). These hybridization patterns indicate that, on all NOR-bearing chimpanzee chromosomes, DJ-like sequences are located immediately proximal to rDNA. As we have sequenced across the boundary between DJ-like sequences, we know the directionality of rDNA repeats. Moreover, we conclude that rDNA and abutting DJ-like sequences are inverted as a unit, in comparison to humans, on all chimpanzee acrocentrics and that rDNA is transcribed in a centromeric to telomeric direction (Fig. 4D).

Proposed human DJ encoded transcripts, disnor 187 and 238, show 95.4% and 97.4% identity with the chimpanzee “DJ-like” contig. Further underscoring the functional conservation of the DJ sequences on chimpanzee acrocentrics, we can readily detect the chimpanzee equivalent of human disnor 238 by RT-PCR using RNA prepared from chimpanzee cells (Supplemental Fig. S12).

### Structural variation in further distal regions

**Figure 5.**
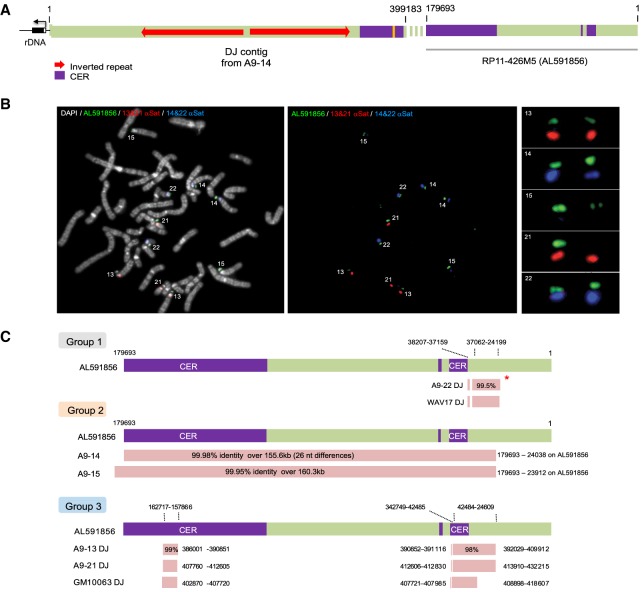
Far-distal regions exhibit variation between acrocentric chromosomes. (*A*) A schematic illustrating the proposed location of sequences present in BAC clone AL591856 relative to the DJ contig from A9-15 (HSA15). (*B*) A normal human female (F5) metaphase spread probed with BAC clone AL591856 (green) and alpha satellite probes recognizing HSA13/21 (red) and HSA14/22 (far red, pseudocolored here in blue). Chromosomes were DAPI-stained (pseudocolored in gray). Enlarged versions of all 10 acrocentric p-arms are shown on the *right*. (*C*) Human acrocentric chromosomes present in hybrid lines show varying composition of AL591856-like sequences in far-distal regions. The portions of DJ contigs and separate contigs that have high sequence identity with far-distal BAC clone AL591856 are shown below a schematic representation of this BAC. These fall into the same three group classifications described in Figure 3C. Coordinates and perent identities are indicated. The red asterisk beside group 1 member A9-22 indicates the presence of a second contig with lower, but still significant, homology (∼87%) with AL591856 (see Supplemental Fig. S13).

As most sequence variability occurs in the distal ends of DJ contigs (Fig. 2B), we sought to identify further distal sequences. We reasoned that BAC clones with large blocks of CER satellites may contain sequences further distal to the current DJ. We identified BAC clone RP11-426M5 (GenBank: AL591856), which includes a terminal 60 kb of CER satellites with two additional small internal CER blocks (Fig. 5A). FISH performed on metaphase spreads from a human donor reveals that sequences hybridizing to AL591856 are found distal to NORs on acrocentric p-arms. Critically, the strength of hybridization signal was highly variable, suggesting that this probe was capturing significant structural variation (Fig. 5B). In particular, we note absence of, and weak, hybridization signals on single HSA21 and HSA15 chromosomes, respectively. As all acrocentrics have a DJ consensus sequence immediately distal to the rDNA array, we conclude that sequences hybridizing to AL591856 lie further distal. Although the original DJ contig and AL591856 do not overlap in sequence, the directionality of repeats within the CER satellite block suggests positioning and orientation of AL591856 relative to the DJ contig shown in Figure 5A.

The wide range of hybridization signals observed with the further distal BAC clone, AL591856, on normal human metaphase spreads suggests that structural variation may increase as we move further distal toward the telomere. As the capture probe library also contained oligos derived from the AL591856 (Supplemental Fig. S4B), we could address this issue directly, by examining sequence contigs assembled de novo from postcapture ROIs. We demonstrate that the AL591856-like sequence composition falls into the same three groupings identified above by MAAFT of DJ contigs (Fig. 2C).

As described above, group 1 DJ contigs from A9-22 (HSA22) and WAV17 (HSA21) are virtually identical over the last 112 kb. Alignment of these contigs with AL591856 reveals that the final 14 kb of both contigs has 99.5% sequence identity with AL591856, both having an identical internal deletion of ∼100 kb (Fig. 5C). Sequencing of A9-22 (HSA22) identified a second contig of ∼103 kb with blocks of similarity (86%–87% identity) to AL591856 (Supplemental Fig. S13). This contig is absent in WAV17 (HSA21). Sequencing of group 2 members, A9-14 (HSA14) and A9-15 (HSA15), identified sequence contigs of 155 and 160 kb, respectively, that are virtually identical to AL591856 (Fig. 5C). Finally, sequence alignments reveal DJ contigs from group 3 members, A9-13 (HSA13), A9-21 (HSA21), and GM10063 (Xder21), have high sequence homology (98%–99% identity) in their termini with blocks of sequence from AL591856 (Fig. 5C). These findings provide further evidence for exchanges between heterologous acrocentric chromosomes and sequence-based support for the heterogeneity observed in FISH on normal human metaphase with AL591856 (Fig. 5B).

For further confirmation, we generated a probe from the region of BAC AL591856 that is present in A9-14 and A9-15, but missing in A9-13, A9-21, and GM10063. This far-distal (FD) probe is comprised of five cloned PCR products designed to avoid repetitive sequence (Supplemental Fig S14). Metaphase spreads from all the hybrid lines except A9-21 were probed with DJ BAC CT476834 together with the FD probe (Supplemental Fig. S10). As expected, both A9-14 and A9-15 showed strong FD signals. A9-22 exhibited a weak FD signal, presumably due to the AL591856-related contig identified by sequence capture (Supplemental Fig. S13). A9-13, WAV-17, and GM10063 were positive for the DJ but devoid of FD signal. Results from these experiments confirm that differential hybridization signals obtained using BAC clone AL591856 detect genuine differences in sequence composition. This demonstrates that AL591856 is a suitable probe for capturing inter-individual structural variation in NOR far-distal regions.

**Figure 6.**
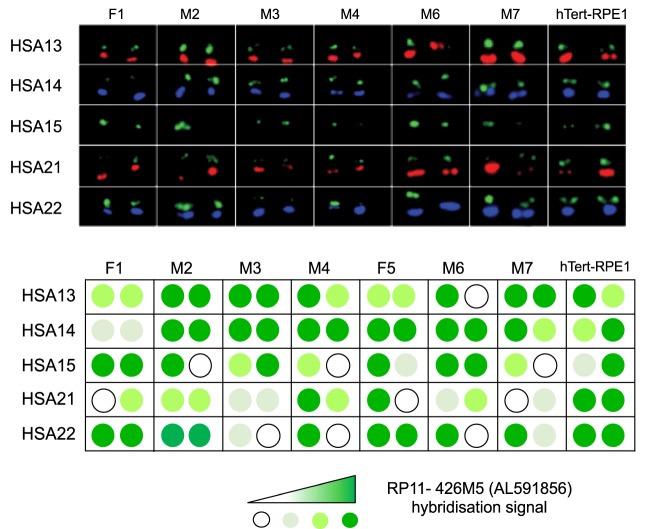
Far-distal regions exhibit variation among human individuals. Metaphase spreads from six human individuals; M (male) and F (female) and hTert-RPE1 cells were probed as above. Shown here are the p-arms of 10 acrocentric chromosomes present in a single representative spread from each individual. Full spreads are presented in Supplemental Figure S15. The relative signals from the AL591856 probe in each spread were classified according to an arbitrary four-point scale and shown in tabular format *below*. Results from F5 above were also included.

Metaphase spreads from a further six human donors were analyzed using the same probing scheme as in Figure 5B. Relative AL591856 hybridization signals from the acrocentric p-arms present in all seven individuals and cell line hTert-RPE1 were classified on a four-point scale ranging from zero to maximal hybridization signals (Fig. 6; Supplemental Fig. S15). Around 60% of acrocentric p-arms show strong hybridization signal, like group 2 chromosomes from A9-14 and A9-15. The remaining chromosomes show weak or no hybridization signal, as would be expected for group 1- and 3-like chromosomes. In each of the seven normal individuals, at least one acrocentric, either HSA13, HSA15, HSA21, or HSA22, is devoid of AL591856 signal. We conclude that FISH, with the BAC AL591856 probe, detects dynamic structural variation between human individuals in the sequence composition of NOR distal regions on acrocentric chromosomes.

## Discussion

Similarities among the NOR-bearing p-arms of the five human acrocentric chromosomes demand novel approaches in determining their DNA sequence. Here, we exploited a panel of monochromosomal somatic cell hybrids that retain intact and functional NORs to examine each of these chromosomes in isolation. Combining sequence capture and long-read sequencing, we could deconvolve NOR distal sequences from all five acrocentric chromosomes, including two additional versions of HSA21. Analyses of these sequences, now incorporated into human genome reference GRCh38.p13, reveals three major findings. First, DJ sequences exhibit both sequence and functional conservation among human acrocentrics and during our recent evolutionary history. Secondly, we provide evidence for recombination events between heterologous acrocentrics occuring within the DJ region and involving exchanges of further distal regions, possibly including the remainder of the chromosome arm. Finally, we uncover dynamic structural variation in acrocentric p-arms between human individuals.

Comparison of DJ contigs from the seven chromosomes analyzed confirm that very similar sequences are present on all human acrocentric chromosomes. The first ∼300 kb that encompass the inverted repeats are virtually identical on all the acrocentric chromosomes, except for the presence of indels up to 5 kb. One might imagine that inverted repeat structure would be genetically unstable and prone to rearrangement or loss, yet not only are they present on every human acrocentric chromosome, they are also present on chimpanzee acrocentric chromosomes. Surprisingly, “DJ-like” sequences, together with rDNA, are in an inverted orientation relative to human on all chimpanzee acrocentrics. Inversions such as this, distributed through the genome, are the most common large-scale sequence differences among humans and great apes (Catacchio et al. 2018). It appears that DJ or DJ-like sequences abutting rDNA arrays are conserved in the human lineage. Our evidence for transcripts originating from DJ-like sequences in chimpanzees implies that conservation extends to transcripts originating from these sequences. Moreover, we show that depletion of DJ transcripts in human cells results in profound nucleolar stress. Precisely how these proposed lncRNAs function remains uncertain and will undoubtedly be a subject for future investigation. The presence of these transcripts in monochromosomal somatic cell hybrids should facilitate future studies, particularly as human NORs can be reactivated in this context. To date, we have been unable to identify DJ-like sequences in nonprimate mammals. This is likely due to the lack of sequence information, as no rDNA junction sequences have yet been described.

It is generally viewed that homogeneity of rDNA repeats across the five human NORs is maintained by exchanges between them. The high level of sequence identity between all seven DJ contigs supports this view. More detailed analysis reveals that precisely identical indels are observed on heterologous acrocentric chromosomes, providing further support. Moreover, the distribution of indels suggests that exchanges between heterologous acrocentric p-arms is not restricted to rDNA arrays but can also occur within the DJ region itself. For example, the distribution of 1.5- and 0.3-kb deletions suggests there have been exchanges between heterologous acrocentrics in the intervening ∼60 kb. In contrast, 0.3- and 5-kb deletions, only ∼11 kb apart, appear to be in linkage disequilibrium.

Alignments of all seven DJ contigs reveal that their most distal regions have a lower overall level of sequence identity. Thus, available DJ contigs can be divided into three subgroups, each showing higher sequence identity within the group. Interestingly, the sequence similarity within a group can be extended further distal toward the telomere. We identified a BAC clone, AL591856, that represents sequences further distal to the DJ on a proportion of acrocentrics. Inclusion into our capture probe design allowed us to demonstrate that high-level sequence identity within group members extended to further distal sequence. For example, group 2 members A9-14 and A9-15, almost identical in the distal ends of their DJ contigs, contain sequences that are virtually identical to each other and to the sequence of BAC AL591856. This indicates that exchanges between heterologous acrocentrics involve large chromosomal regions. Identification of groups 1–3 should not be interpreted as a definitive description of the total variation in the human population. Future sequencing of more individual acrocentrics will be required. The application of optical mapping techniques such as Bionano could also prove valuable (Chan et al. 2018).

Genetic exchanges between homologous chromosomes during meiosis can be mediated by either crossover (CO) or noncrossover (NCO) events. Although NCOs, gene conversions, are an order of magnitude more common than COs, their tract lengths are limited to 1 kb in length (Jeffreys and May 2004). Due to the observed length of genetic exchanges between heterologous acrocentrics, we can infer that they involve COs. The number of COs typically observed in human meiosis between homologous chromosomes is low, averaging 49.1 per male pachytene stage cell (Lynn et al. 2002). Thus, we can speculate that genetic exchanges between heterologous acrocentric p-arms involve the transfer of chromosome ends.

Exchanges between heterologous acrocentrics and ensuing structural genomic variation between human individuals cannot be studied using current sequencing technologies. Sequencing reads cannot be mapped to any single one of the 10 acrocentric p-arms represented in a genomic DNA sample. To circumvent this, we have used BAC clone AL591856 in FISH on metaphase spreads from a number of individuals. The results we obtained reveal dynamic structural variation in these distal regions of acrocentric p-arms. Our interpretation is that this variation, of the type detected in the monochromosomal hybrids, arises from heterologous exchanges. A previous study has suggested that rDNA clusters are subject to meiotic rearrangement at a frequency >10% per cluster per meiosis (Stults et al. 2008). However, exchanges between homologous or heterologous chromosomes could not be distinguished. Our study confirms that exchanges between heterologous chromosomes can occur and suggests that they may be more frequent than previously anticipated.

In most eukaryotes, meiotic crossovers are essential for error-free chromosome segregation but are specifically repressed near centromeres, by a mechanism involving heterochromatin, to prevent missegregation (Nambiar and Smith 2016). It is possible that genetic exchanges between heterologous acrocentric p-arms are similarly repressed. It is interesting to speculate that exchanges may instead occur outside of meiosis—for example, in early embryos where epigenetic marks underpinning constitutive heterochromatin are not yet established (Fadloun et al. 2013). Indeed, the high levels of recombination within rDNA arrays, observed in cancer cells (Stults et al. 2009), with deregulated epigenetic processes, may similarly involve exchanges between heterologous acrocentrics. Further sequencing efforts should guide design of next-generation FISH probes that will facilitate future research into when and how frequently exchanges between heterologous acrocentrics occur.

As rDNA arrays and DJ sequences remain linked together despite dynamic structural variation in human acrocentric chromosomes and over evolutionary time, we should now consider NORs in molecular terms as being comprised of rDNA arrays and linked DJ or DJ-like sequences. Information on rDNA distal and proximal sequences in other mammals, currently missing, would help confirm or rule out this hypothesis. The dynamic structural variation among acrocentric p-arms that we have revealed also provides a rationale for localizing NORs on multiple dedicated chromosome arms. Frequent exchanges between these chromosome arms will have minimal impact on the remainder of the genome—specifically, the chromosomal regions containing the approximately 21,000 protein coding genes. Finally, the dynamic structural variation we have described here poses difficulties regarding how to incorporate acrocentric p-arms into the human genome reference. Once we have a more complete description of the variation, it may be best to include all possible variants as alternative loci without attribution to any single acrocentric chromosome.

## Materials and methods

### Plasmids and BACs

The human TAF_I_A open reading frame (orf) was cloned into the Gateway entry vector pENTR/D-TOPO, and TAF_I_B-D orfs were individually cloned into pENTR4. Using Gateway recombination, cloning orfs were transferred to mammalian expression vector pcDNA6.2 nLumio-DEST (Thermo Fisher Scientific). All BACs used in this study (Supplemental Table S2) were obtained from the BACPAC Resource Center (Children's Hospital Oakland Research Institute, Oakland, California).

### Cell culture

A panel of monochromosomal somatic cell hybrids (A9-13, A9-14, A9-15, A9-21, and A9-22) containing individual human acrocentric chromosomes derived from a normal male fibroblast cell line, 1BR.2, has been described and characterized previously (Cuthbert et al. 1995; Sullivan et al. 2001). They were cultured in Dulbecco's modified Eagle's medium (DMEM) supplemented with 10% fetal calf serum (FCS) and 400 U/mL hygromycin B. Monochromosomal somatic cell hybrid lines containing intact HSA21, WAV17, and the der(X)t(X;21)(p21;p12) reciprocal translocation products, GM09142 and GM10063, were obtained from the Coriell Institute and cultured as specified. Sequence tagged sites (STSs) used to confirm the identity of the human chromosome in each of the monochromosomal somatic cell hybrids were as follows: HSA13, WI-5823 (G04979.1); HSA14, W-4541 (G03458.1); HSA15, WI-3162 (G04204.1); HSA21, WI-5424 (G03676.1); HSA22, WI-4572 (G03792.1); HSAX, DXS453 (X54600.1). Primer sequences and PCR conditions were obtained from each GenBank accession (shown in parentheses above). hTERT-RPE1 cells were maintained in DMEM/nutrient mixture F-12 Ham containing 2 mM L-glutamine, 10% (v/v) FBS, and 0.25% (v/v) sodium bicarbonate. Their identity was confirmed by genotyping (Eurofins). Chimpanzee (*P. troglodytes*) B-cell lines from named individuals, Vanessa (female), and Zeef and Walter (males), were obtained from the Biomedical Primate Research Centre (Netherlands) and cultured in RPMI 1640 (+L-glutamine, +25 mM HEPES), 10% FCS, Pen/Strep, and 7.5 µg/mL Tenofovir (Selleckchem). hTert-RPE1 cells stably transfected with mCherry-Cdt1 and mAG-Geminin were grown in the above media, supplemented with 300 µg/mL G418 and 5 µg/mL blasticidin.

### FISH on cells and chromosome spreads

FISH experiments on metaphase chromosome spreads were performed as previously described (Mais et al. 2005; van Sluis et al. 2016). Normal human metaphase slides from seven donors (five male and two female) were obtained from Applied Genetics Laboratories. In RNA-FISH experiments, cells were grown on Superfrost Plus microscopy slides. After removal of media, slides were rinsed with ice-cold 1× PBS. Subsequent washes were performed on ice. Cells were permeabilized with cytoskeletal buffer (CSK; 100 mM NaCl, 300 mM sucrose, 3 mM MgCl_2_, 10 mM HEPES, 0.5% Triton X-100) supplemented with 1:1000 dilution of Vanadyl RNase inhibitor (Sigma) for 5 min. Following PBS washes and fixation with 4% PFA, slides were washed with 70% ethanol. Slides could be stored in 70% ethanol at −20°C for up to1 mo. To dehydrate the slides, they were passed through an ethanol series of 80%, 95%, and 100% for 3 min each. Slides were then dried at 42°C and combined with denatured hybridization probe(s) in Hybrisol VII. DNA and RNA FISH probes were directly labeled by nick-translation using either Green 496 dUTP, Red 580 dUTP, or Far Red 650 dUTP (Enzo). Human rDNA was visualized with a 12-kb EcoRI restriction fragment from the inter-genic spacer, located immediately upstream of the gene promoter. The human 5′ ETS RNA-FISH probe is a 2.9-kb Not I restriction fragment (+270/+3170), and the mouse 5′ ETS RNA-FISH probe is a 2.9-kb AcuI-SalI fragment (+120/+3100); coordinates refer to Genbank accessions U13369 (human) and BK000964 (mouse). For generation of chromosome-specific alpha satellite probes, degenerate primers AlphaSat1 and 2 (Supplemental Table S3) were used in PCR with genomic DNA prepared from monochromosomal hybrids as template. Following a secondary amplification with the same primers, PCR products were directly labeled by nick-translation. HSA13 and 21 probes cross react but metaphase chromosomes can be easily distinguished based on size. HSA14 and 22 probes also cross-react and are distinguished using the same rationale. BAC clones used as FISH probes were prepared using NucleoBond Xtra (Macherey-Nagel) and labeled as above.

### Microscopy

Images were captured and merged using a Hamamatsu Orca Flash 4.0 V2 camera and LASX 2.0 software (Leica) with a 63× Plan Apochromat objective mounted on a Leica DMi8 imaging microscope illuminated by a Lumencor Spectra X Fluorescence Light Source. Typically, Z-stacks from the bottom to the top of a cell or metaphase spread were taken. The number and distance between the stacks was system-optimized. Image stacks were deconvolved by a blind method using auto-generated point spread functions (PSFs) with 10 iterations in LASX 3.3 software containing the 3D deconvolution module (Leica). Background was removed and intensity rescaled. For publication purposes, images were contrast-enhanced, but the original data was retained. Images shown are maximum projections.

### Transfections and antisense depletion experiments

Monochromosomal somatic cell hybrids were transfected with an equimolar mixture of all four TAF_I_ expression plasmids using Lipofectamine 2000 (Thermo Fisher Scientific). At 72 h posttransfection, cells were fixed and subjected to RNA FISH. In antisense experiments, 2 × 10^6^ hTert-RPE1 cells were electroporated with 800 pmol of either ASO1 or ASO1M using either Amaxa Nucleofector and Nucleofection Solution V (Lonza) or a Neon electroporation system (ThermoFisher). Cells were plated out and counted at 24-h intervals.

### Reverse transcription PCR

First-strand cDNA was synthesized using ProtoScript Reverse Transcriptase (NEB) with either oligo-dT or random hexamer priming. PCR was performed on cDNA using primers described in Supplemental Table S3. In all cases, cDNA reaction mixes without added reverse transcriptase served as negative controls.

### Transcription and replication assays

Cells were incubated with 1 mM EU or 10 µM EdU (Berry & Associates, Inc.) for 30 min. Coverslips were fixed with 4% (w/v) PFA/PBS, and cells were permeabilized with 0.5% (v/v) Triton X-100/PBS. The click chemistry reaction was assembled with 10 mM sodium L-ascorbate (Sigma), 0.1 mM Biotin-TEG-azide (Berry & Associates, Inc.), and 2 mM Cu(II)SO4 (Sigma) in PBS. This was incubated at RT in the dark for 30 min. Following PBS washes, EU slides were incubated with primary antibodies against UBF and Nop52 as previously described (van Sluis and McStay 2015). Sites of synthesis were visualized with 1:200 Streptavidin conjugated TexasRed (Rockland) or Alexa647 (Jackson ImmunoResearch) for EU and EdU, respectively, combined with secondary antibodies (Jackson ImmunoResearch), diluted in 1% BSA/PBS for 1 h at 37°C.

### Precapture library preparation

Covaris gTUBEs were used to shear high molecular weight genomic DNA prepared from hybrid cell lines. For each line, 30 µg of genomic DNA in 150 µL TE buffer was loaded onto a gTube and subjected to six cycles of centrifugation (12 spins, 30 sec/spin) at 13,200 rpm in a microfuge (Eppendorf). The bulk of sheared DNA was between 3 and 8 kb in length. Further size selection was performed, initially using preparative agarose gel electrophoresis, but more commonly with a Blue Pippin (Sage Science). For Blue Pippin based size selection, 5-µg aliquots of gTUBE sheared DNA were electrophoresed in each lane of a 0.75%, dye-free agarose gel cassettes alongside an external marker. The instrument was programmed to select DNA in either the 3- to 5-kb or 4- to 6-kb size range. Recovered DNA (typically at 40 ng/µL in electrophoresis buffer) was used directly for precapture library preparation. First, 200 ng of size-selected DNA were end-repaired and A-tailed, and indexed SeqCap EZ Adapters (Roche) were ligated using a KAPA Hyper Prep kit according to a protocol provided by Roche/Pacific Biosciences. Following adapter ligation, DNA was recovered using 0.8× volume of AMPure XP beads (Beckman Coulter) and eluted in 50 µL Elution Buffer (EB, 5 mM Tris pH 8.5). Twenty-five microliters of the purified adapter ligated product were amplified using KAPA HiFi polymerase in a total volume of 200 µL containing 40 µL KAPA HiFi Fidelity Buffer (5×), 6 µL KAPA dNTP Mix (10 mM), 12.8 µL of primers (SeqCapPB1 and 2 each at 12 µM), and 4 µL KAPA HiFi Enzyme (1 U/µL). Note that primers contained phosphorothioate linkages at their 3′ ends to resist the 3′–5′ exonuclease activity of the HiFi polymerase (Supplemental Table S3). The reaction was divided into four 50-µL reactions, and PCR was performed as follows: 95°C (2 min), 10 cycles of 95°C (20 sec)/65°C (20 sec)/72°C (5 min), and one cycle of 72°C (5 min). PCR products were recovered using 0.8× volume of AMPure XP beads and eluted in 50 µL EB. Typically, the final concentration of the precapture library was in the range of 100–200 ng/µL. For quality control, size distribution of DNA was determined using either agarose gel electrophoresis or an Agilent Bioanalyzer DNA12000 chip. DNA concentrations were determined using both a Picodrop spectrophotometer and a Qubit fluorimeter to distinguish double- and single-stranded DNA.

### Sequence capture and postcapture library preparation

Sequence capture was performed using a protocol developed by Roche/Pacific Biosciences with SeqCap reagents supplied by Roche. Two micrograms of precapture library was combined with 5 µg of a 19:1 mix of mouse and human Cot-1 DNA and 1 µL each of the universal and the appropriate index blocking oligonucleotides (1000 µM SeqCap HE Universal and 1000 µM SeqCap HE Index, respectively). The DNA sample was then dried using a speed vacuum. Ten microliters of 2× Hybridization Buffer and 4 µL of Hybridization Component A were added to the dried DNA, and the tube was incubated at 95°C for 10 min to denature the target DNA. After a brief spin, the sample was transferred to a 0.2-mL PCR tube containing 3 µL of water and 3 µL of the custom EZ capture library, then incubated for 16–20 h in a thermocycler with heated lid. Note that the amount of EZ capture library used was half of that recommended by the manufacturer, due to the relatively small size of the target. Capture with Dynabeads M-270 Strepatavidin (Thermo Fisher Scientific) and postcapture washes were performed exactly as described in the protocol provided by Roche/Pacific Biosciences. The washed beads were suspended in 50 µL of EB and used directly in 200 µL postcapture PCR (4× 50 µL) exactly as described in precapture library preparation, except that 20 cycles were performed. PCR products were recovered using 0.8× volume of AMPure XP beads and eluted in 50 µL EB. Typically, the final concentration of the postcapture library was in the range of 100–300 ng/µL.

### Sequencing of postcapture libraries

Postcapture libraries (2- to 5-µg samples) were sent to the Centre for Genomic Research at the University of Liverpool, where SMRTbell adapters were added and sequencing was performed using a PacBio RS II System with P6/C4 chemistry. In most cases, libraries were sequenced using a single SMRT cell. However, in a few cases two SMRT cells were used.

### Processing of sequencing data and de novo assembly

Reads of insert were generated using the RS_ReadsOfInsert.1 protocol from the PacBio SMRTportal software (v2.3) with Minimum Full Passes set to 3 and Minimum Predicted Accuracy set to 90. Illumina adapter sequences were removed from both the 5′ and 3′ ends of the ROI using cuptadapt (v1.14), trimming 64 bp from each end. BLASR (Chaisson and Tesler 2012) was used to evaluate sequencing coverage by aligning trimmed ROIs to manually curated builds of each acrocentric DJ region. De novo assembly was performed using the Canu assembler (v1.6) (Koren et al. 2016). The trimmed ROIs were used as input, and assembly was performed under default PacBio conditions where the correctedErrorRate is set to 4.5%. In order to optimize contig assembly through repetitive sequence blocks, an additional assembly was performed with the correctedErrorRate of 1%. BLAST-2.6.0_2+ was used to evaluate sequence gaps by aligning Canu contigs for each acrocentric DJ region against manually curated DJ builds. A custom python script was used to analyze BLAST results files to identify the 50- to 200-bp regions that were not covered in the sequence capture.

### Gap filling

Gaps were identified, based on alignments of sequence capture contigs with the original BAC-derived sequence contig described previously (Floutsakou et al. 2013). Targeted PCR was performed using the appropriate hybrid cell genomic DNA as template with the primer pairs shown in Supplemental Table S3 and Q5 High-Fidelity DNA Polymerase (NEB). In most cases, PCR products were sequenced directly using both forward and reverse PCR primers. In some cases, including across large HERV-K integrations and chimpanzee rDNA/DJ junctions, PCR products were cloned into pJET1.2/blunt (Thermo Fisher Scientific) prior to double-stranded sequencing. Primer sequences are shown in Supplemental Table S3.

### Sequence data

GenBank accession codes and links to the Sequence Read Archive (SRA) are listed in Supplemental Table S4.

## Supplementary Material

Supplemental Material
