# Supplementary material for: Human NORs, comprising rDNA arrays and functionally conserved distal elements, are located within dynamic chromosomal regions
Source: Genes Dev. 2019 Dec 1;33(23-24):1688–701. doi: 10.1101/gad.331892.119 (PMC6942050; doi:10.1101/gad.331892.119)
Supplement: Supplemental Material [file supp_33_23-24_1688__index.html]

Human NORs, comprising rDNA arrays and functionally conserved distal elements, are located within dynamic chromosomal regions — Supplemental Material 

# Human NORs, comprising rDNA arrays and functionally conserved distal elements, are located within dynamic chromosomal regions

## Supplemental Material

- Supplemental\_Data.pdf
