# Supplementary material for: Human NORs, comprising rDNA arrays and functionally conserved distal elements, are located within dynamic chromosomal regions
Source: Genes Dev. 2019 Dec 1;33(23-24):1688–701. doi: 10.1101/gad.331892.119 (PMC6942050; doi:10.1101/gad.331892.119)
Supplement: Supplemental Material [file supp_gad.331892.119_Supplemental_Data_.pdf]

Human NORs, comprising rDNA arrays and functionally conserved distal elements, are located within dynamic chromosomal regions

Marjolein van Sluis, Michael Ó Gailín, Joseph G. W. McCarter, Hazel Mangan, Alice Grob and Brian McStay.

Supplementary Information

Figures S1-S15

Tables S1-S4

A

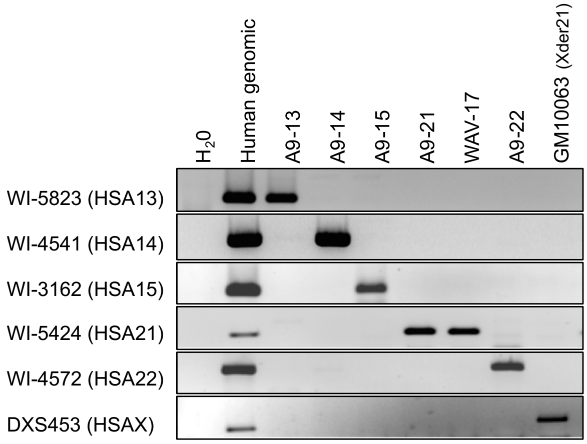

B

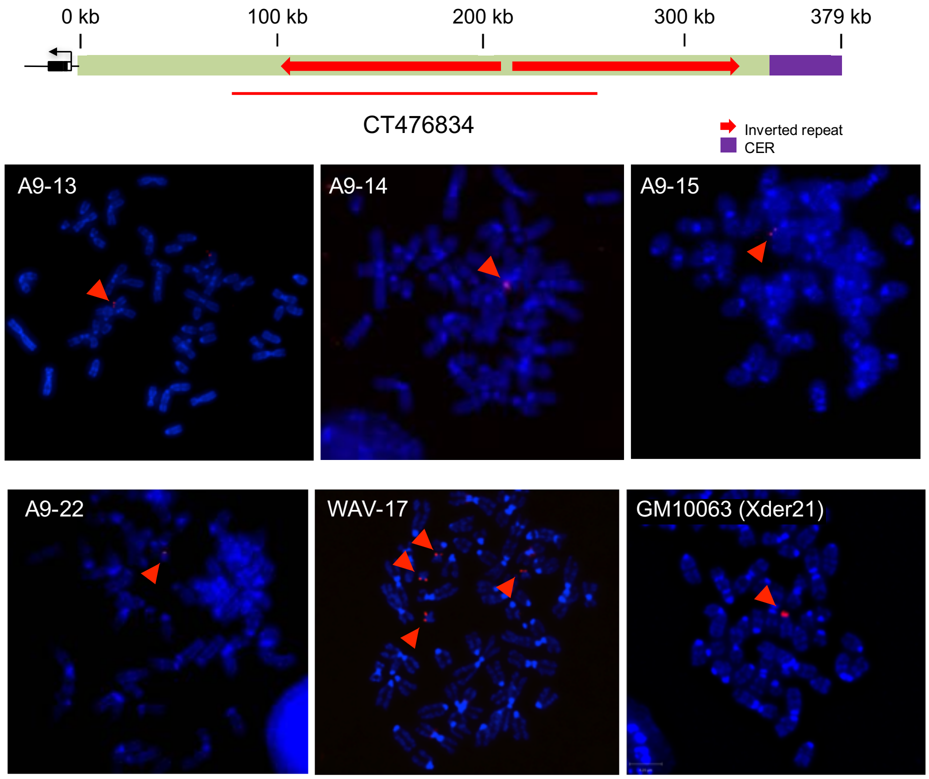

Figure S1. Verification of mono-chromosomal somatic cell hybrid panel. **(A)** PCR amplification of sequence tagged sites (STSs) from all five acrocentrics and HSAX was performed across the hybrid panel. Human genomic DNA and water served as a positive and negative controls respectively. **(B)** Metaphase spreads from the hybrid lines indicated were probed with DJ BAC clone CT476834 (red). Hybridisation signals are indicated by red arrowheads.

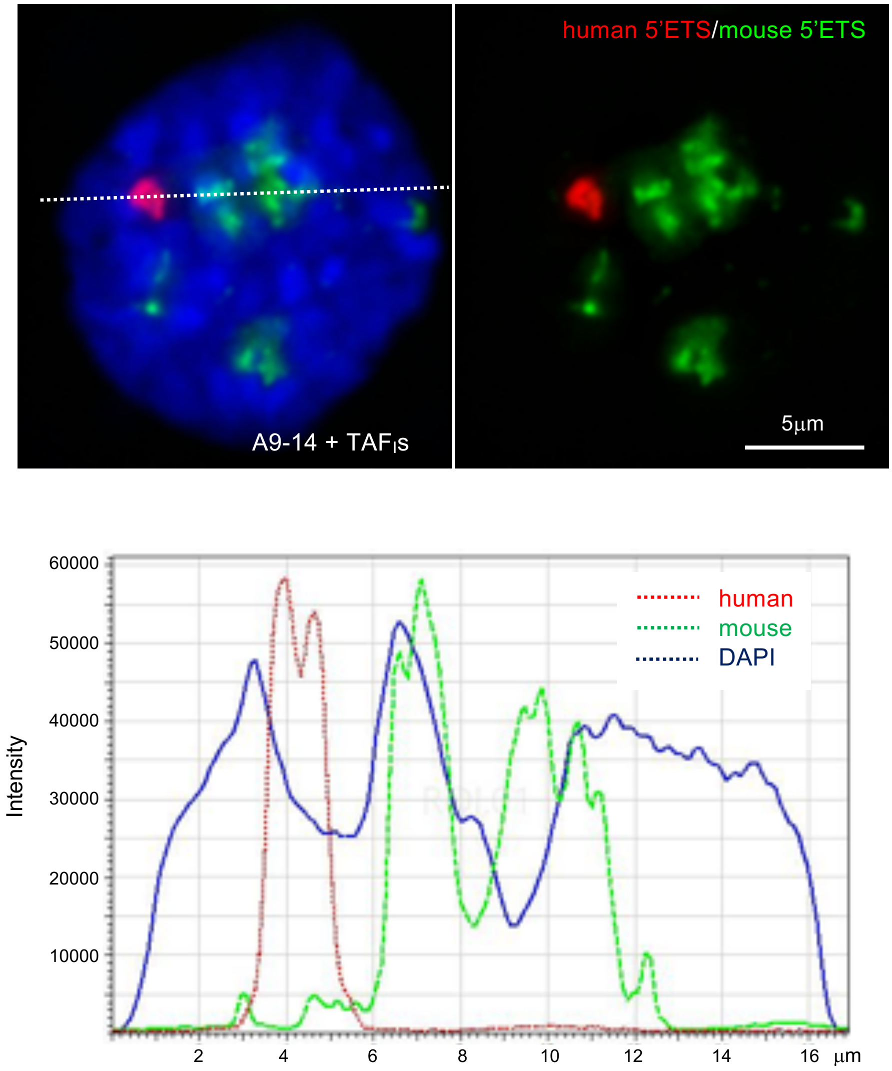

Figure S2. Quantitation of human NOR reactivation. A9-14 hybrid cells were transfected with plasmids encoding human TAF$_I$s. Endogenous mouse pre-rRNA and pre-rRNA from the reactivated human NOR were detected using 5’ ETS probes, green and red respectively. Green and red hybridization signals were captured using identical exposure settings. Fluorescence intensity profiles of DAPI, Red and Green channels across a nucleus, indicated by the dotted white line, are shown below.

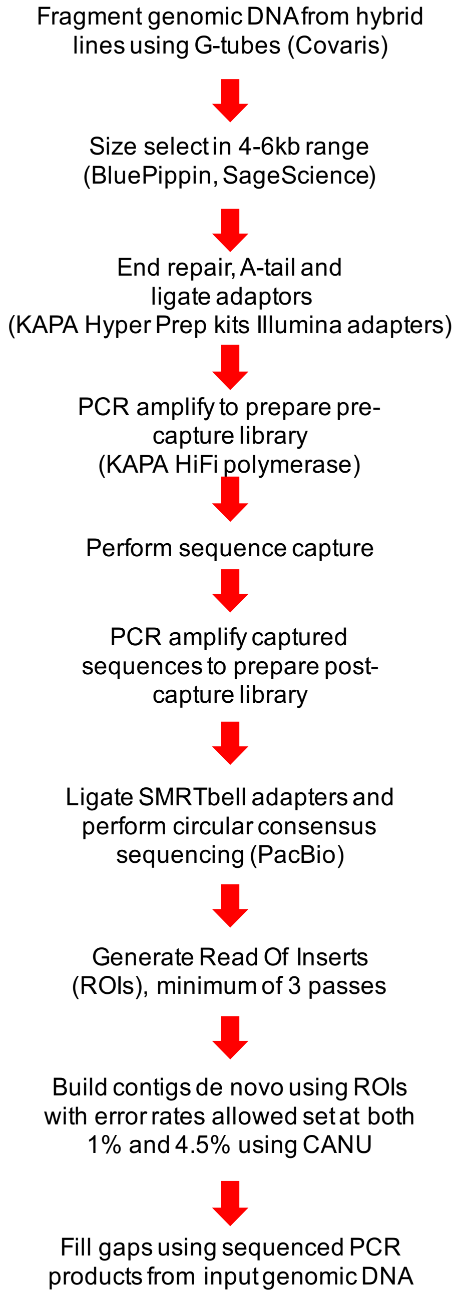

Figure S3. A diagram illustrating the DJ sequencing workflow.

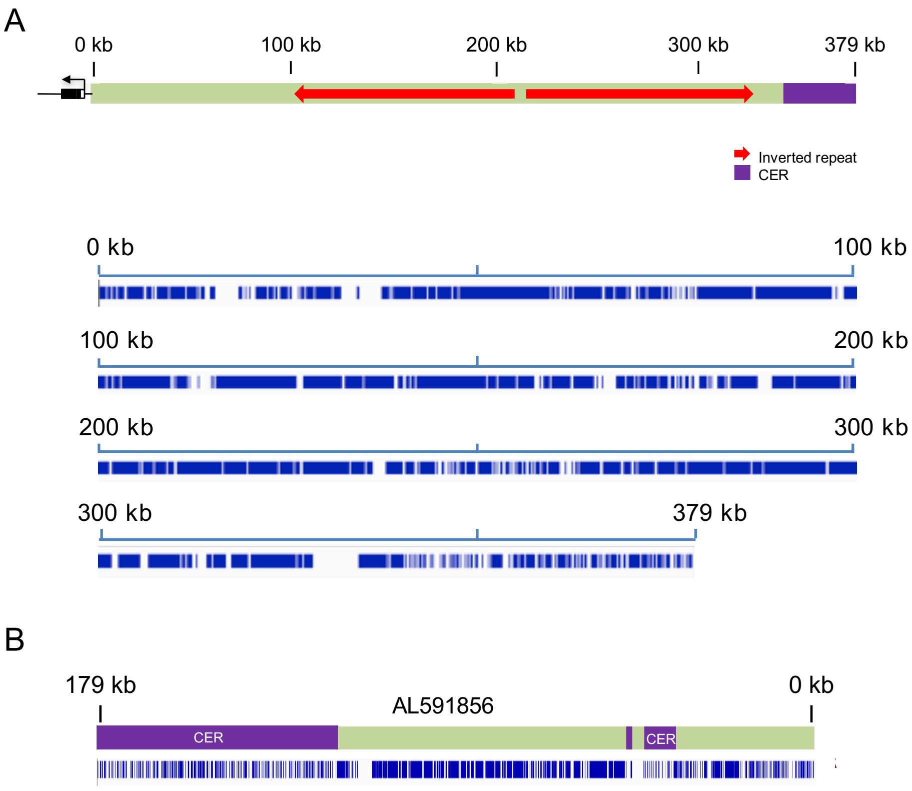

Figure S4. Capture oligo probes. Schematic representations of the original 379 kb DJ contig **(A)** and far distal BAC clone AL591856 **(B**) with the distribution of capture oligos (blue dashes) shown below each.

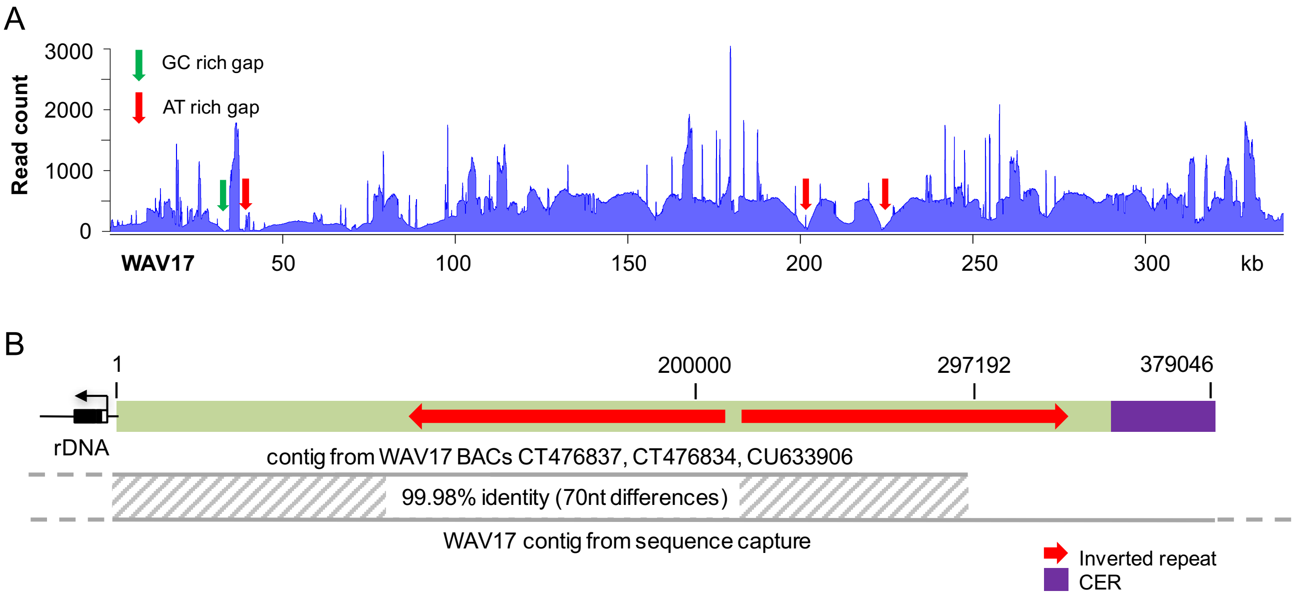

Figure S5. Validation of sequencing strategy. **(A)** Coverage of reads of inserts (ROIs) mapped onto the original DJ contig. The positions of AT and GC rich gaps (50-200 nucleotides) are indicated by red and green arrowheads respectively. **(B**) A comparison of the de novo assembled contig obtained from the WAV17 hybrid with the portion of the original DJ contig that was assembles using BAC clones from WAV17 library. The percentage identity and number of nucleotide differences are indicated.

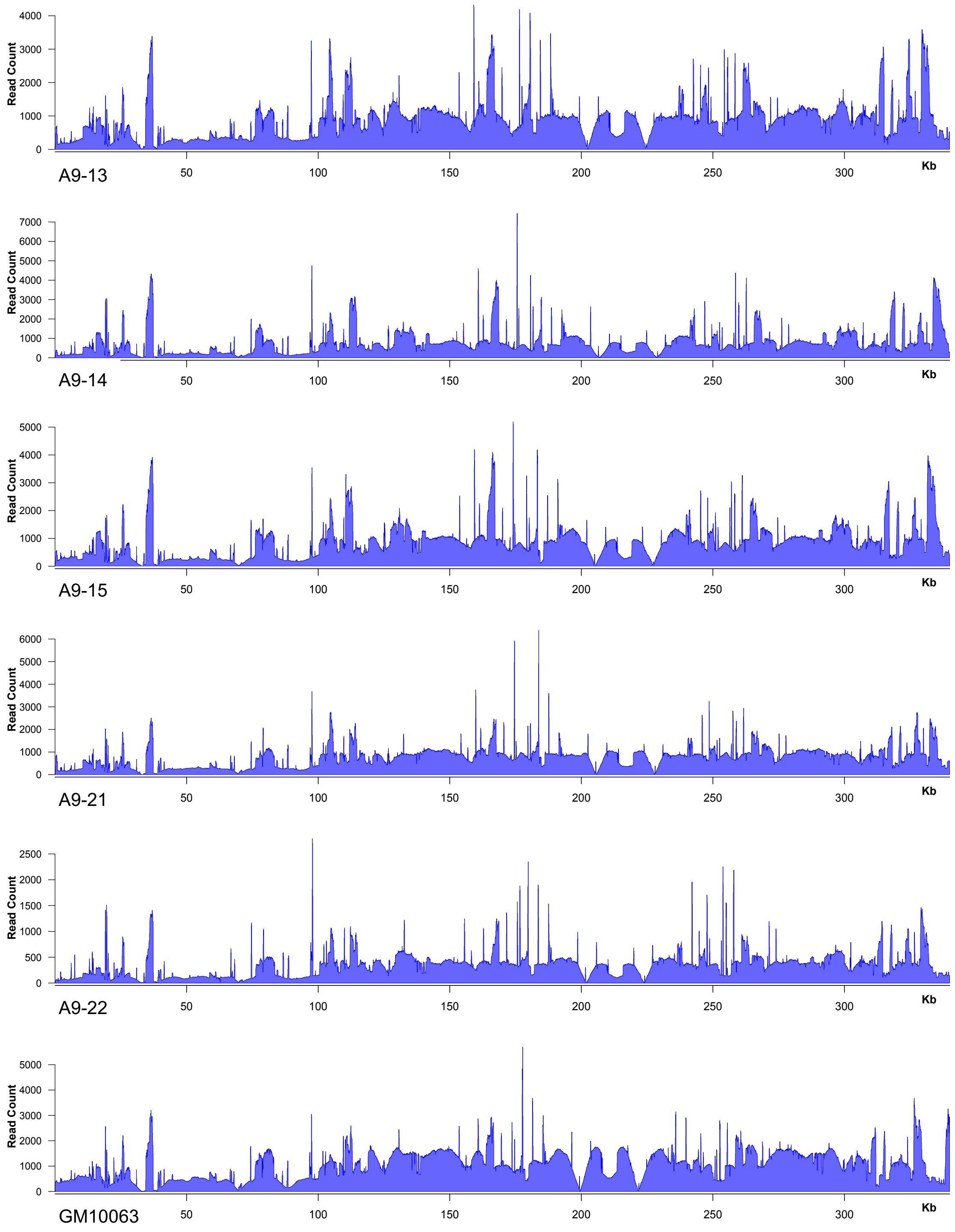

Figure S6. Coverage of ROIs from the remaining 6 hybrids mapped onto the original DJ contig.

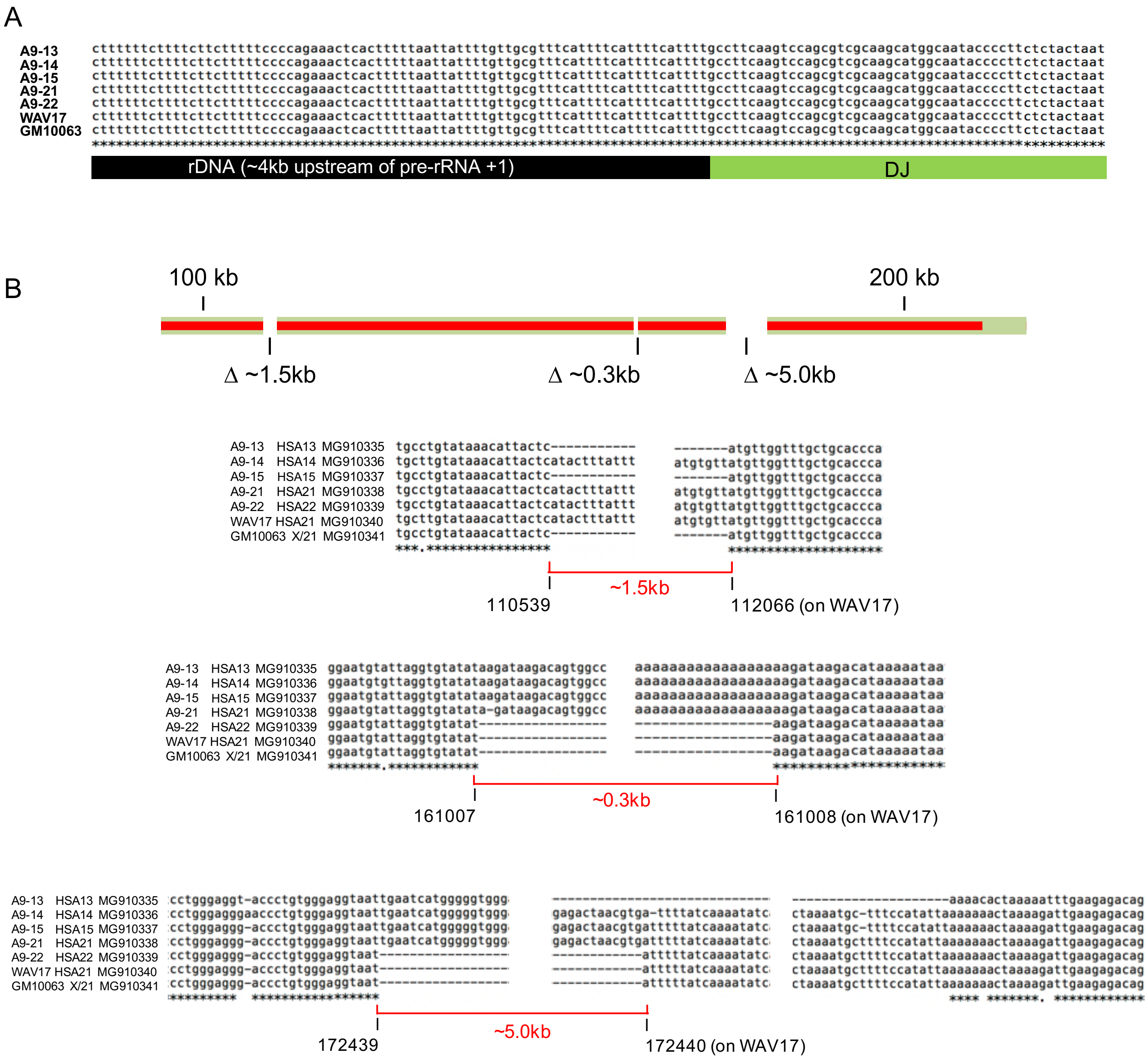

Figure S7. Sequence comparisons of DJ contigs. **(A)** Alignments of sequences at the junction between rDNA and the DJ. **(B)** A schematic representation of three indels observed on the left-arm inverted repeat. Sequence alignment across these indels are shown below. Note that the A9-13 DJ contig has a smaller indel that overlaps the 5kb indel.

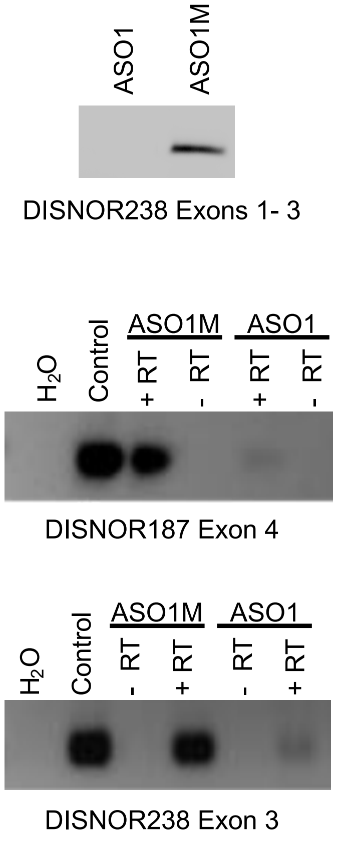

Figure S8. Depletion of DJ transcripts with antisense oligos. RT-PCR performed was performed on RNA isolated from hTert-RPE1 cells treated with either ASO1 or control ASO1M antisense oligos. In the upper panel disnor 238 is examined using exon1 and 3 forward and reverse primers respectively. In the middle panel, forward and reverse primers are from exon 4 of disnor 187. In the bottom panel, forward and reverse primers are from exon 3 of disnor 238. RNA from non-treated cells served as a positive control. Water and reverse transcriptase negative reactions provided negative controls.

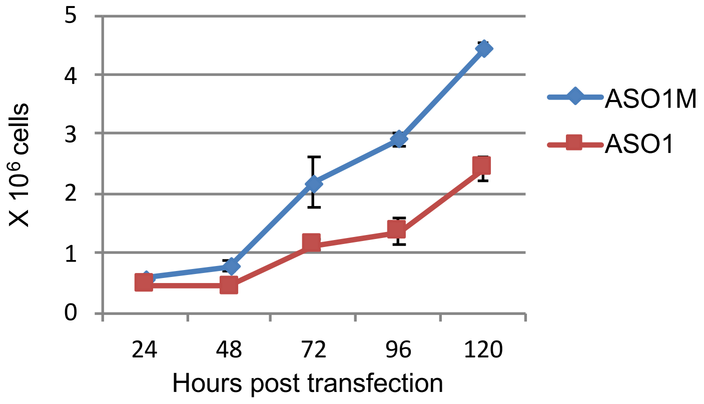

Figure S9. Growth curves after electroporation of hTert-RPE1 cells with ASO1 targeting disnors 187 and 238. ASO1M served as a negative control. Three independent replicates were performed.

A

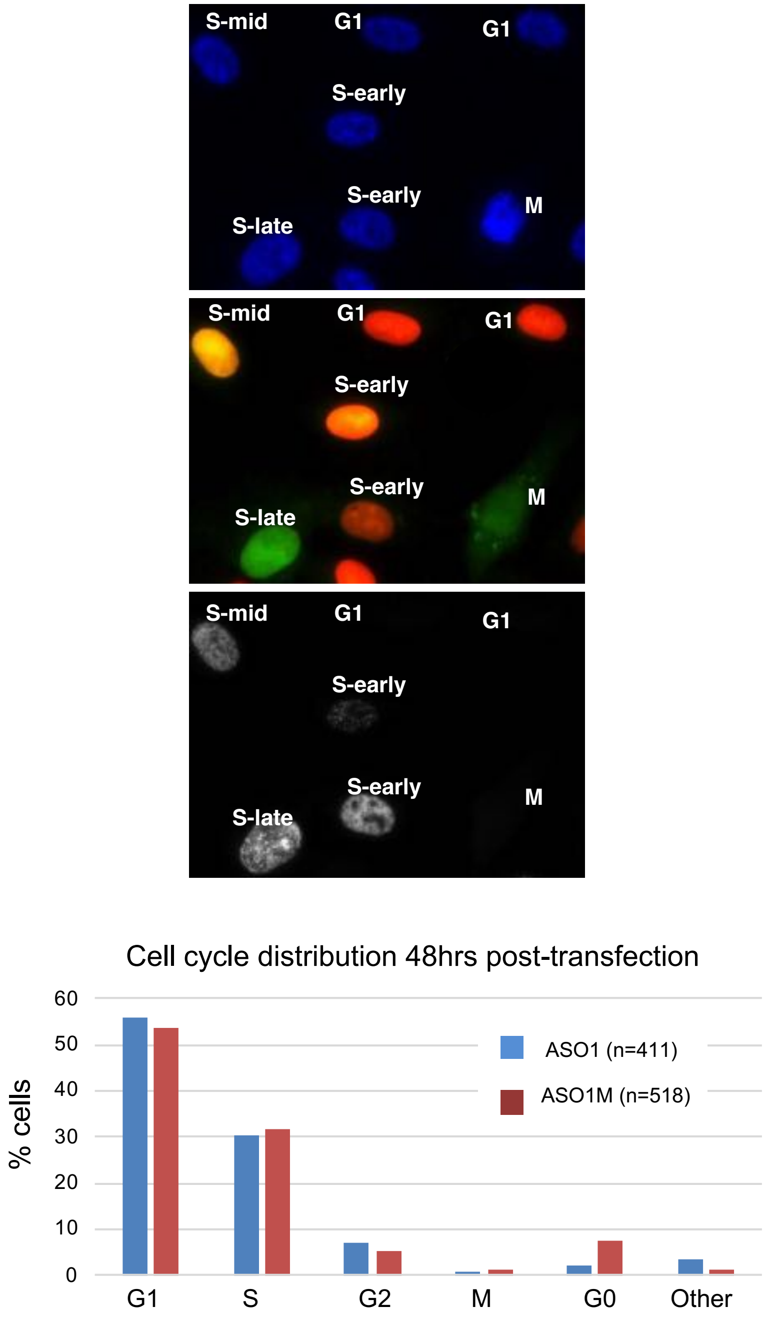

Figure S10. Cell cycle distribution post ASO transfection. **(A)** hTert-RPE1 cells, stably transfected with FUCCI markers mCherry-Cdt1 and mAG- Geminin, were transfected with either ASO1 or ASO1M. 48 hours post transfection cells, were labelled with EdU for 30 mins followed click-chemistry. A field of ASO1M transfected cells is shown and various cell cycle stages are indicated. M-phase cells are recognized by DAPI staining. **(B)** The cell cycle distribution in ASO transfected cells determined by examination of FUCCI markers and EdU incorporation. Note that G0 cells are identified by lack of FUCCI markers. Cells in the columns labelled 'other' express both FUCCI markers (yellow) but lack EdU incorporation, likely reflecting activation of an intra-S-phase checkpoint.

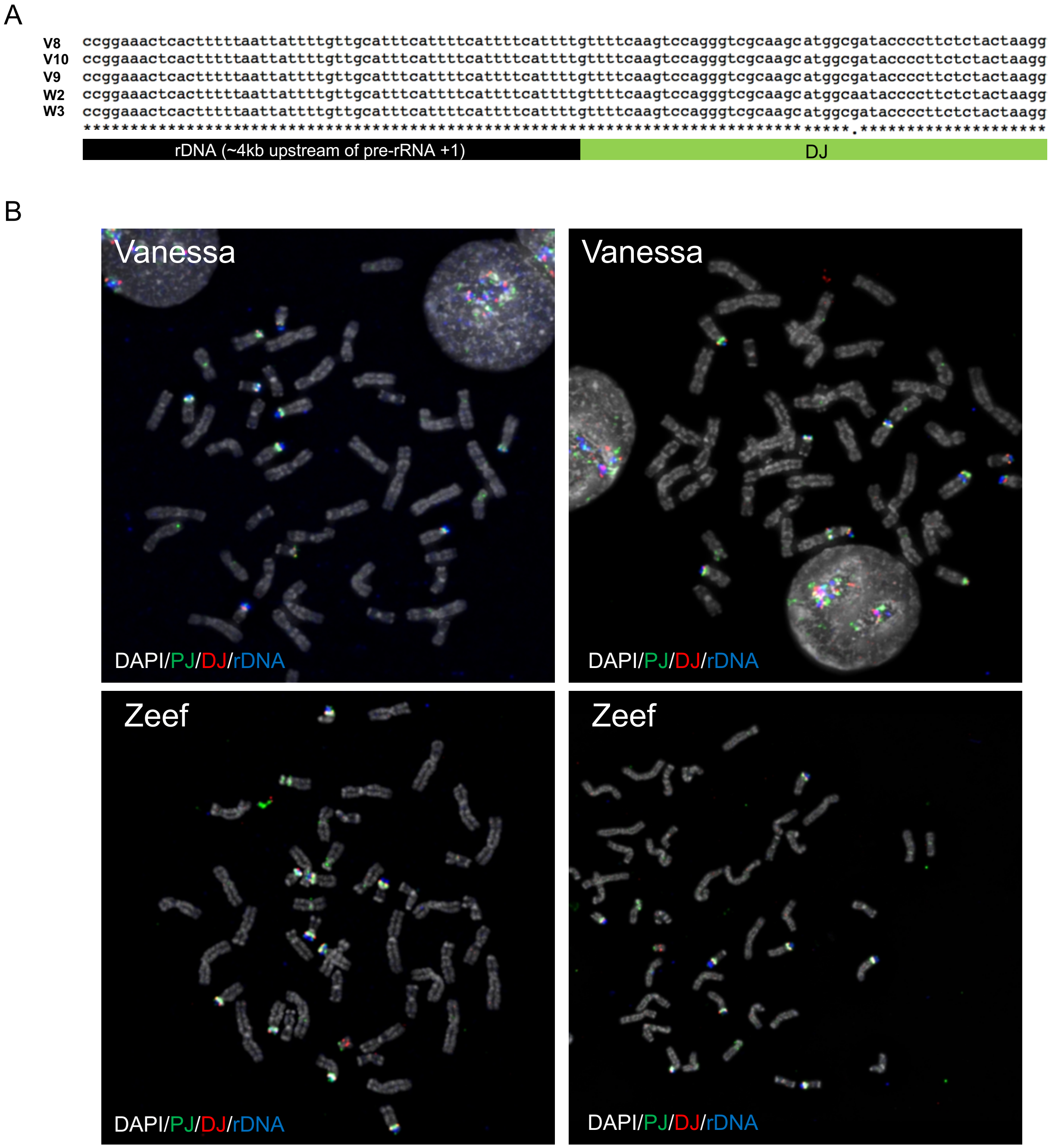

Figure S11. DJ-like sequences in chimpanzees. **(A)** Sequence alignments of rDNA-DJ like junctions. V8,9 and 10 are three independent PCR cloned products from chimpanzee Vanessa and W2 and 3 are from Walter. **(B)** Metaphase spreads from chimpanzees Vanessa (female) and Zeef (male) probed with 'DJ like' chimpanzee BAC clones (red), human PJ BAC clone bP-2154M18 (CR381535) (green) and rDNA (far red, pseudo-coloured here in blue). Chromosomes were DAPI stained (pseudo-coloured in grey).

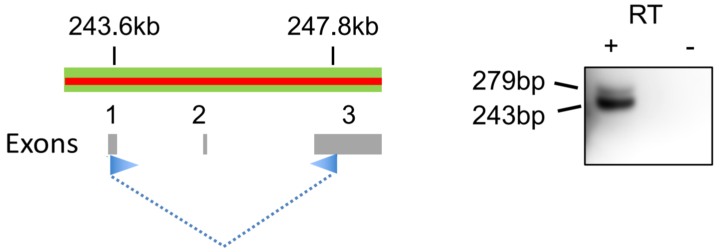

Exon 1 **ACAGCCCTTGTTGCCCTGCG**ATCTGTAGGTCCTTGGGGACGCATAGTTAA
GGTGCCAGGACATCCTGGAAGCTGGGAAATGATTGTTGCAGTGTCAGGAC
Exon 2 CAAGGTCAAATGAGTTATAGCCAAGTCTACAGTAAGATGTGGCAGTATTC
Exon 3 TGTTTTGAAGCCAGGACCATGATTGGGCGGGGAGACCTTCCTTCACCACG
GGTTTGAGGCTATGGCTAAGT**GGTAAGCCTTGGGGAGATGCAG**

Sequence of 243bp PCR product

Exon 1 **ACAGCCCTTGTTGCCCTGCG**ATCTGTAGGTCCTTGGGGACGCATAGTTAA
GGTGCCAGGACATCCTGGAAGCTGGGAAATGTTAGCATTTCATCATATCA
Exon 2 GTGTGAAGATTTCTTCCAGACCATGGCTGGAGAAGGAAAGAAGGTGTGTT
TTGCCTGATTCAGGGACTATAGAGAGAACCAAGTTCTGCAGGCCTGTCAC
Exon 3 CTAAGTATCAGGGCGGAGAGACCTTCCTTCCCCACGGGTTTGAGACTATG
GCTAAGT**GGTAAGCCTTGGGGAGATGCAG**

Sequence of 279bp PCR product

Figure S12. Identification of DJ transcripts in chimpanzees. Based on alignments between human and chimpanzee DJs, the predicted locations of exons 1-3 of a DJ transcript from the right arm of the inverted repeat are indicated. The location of PCR primers used in RT-PCR are also indicated. Results from RT-PCR reactions using RNA from chimpanzee cells are shown on the right. The major band is a 243bp PCR product. The upper 279bp band is either a splicing variant or a similar transcript arising from a chimpanzee acrocentric with a variant DJ sequence. DNA sequences from cloned 243 and 279bp PCR products are shown below. PCR primer sequences are in bold and exon/exon boundaries are indicated by red arrowheads.

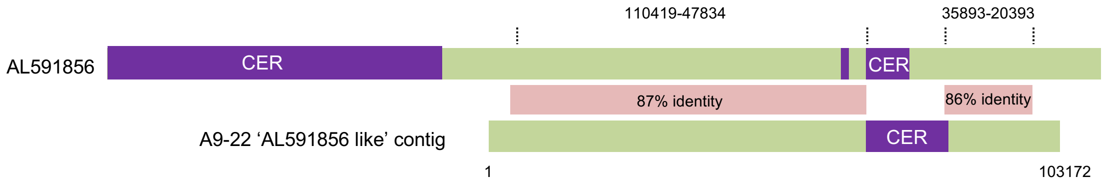

Figure S13. AL591856-like contig in A9-22. A second contig with homology to far distal BAC AL591856 was obtained. A schematic shows this contig positioned below AL591856 with the regions of homology and their percentage identity indicated.

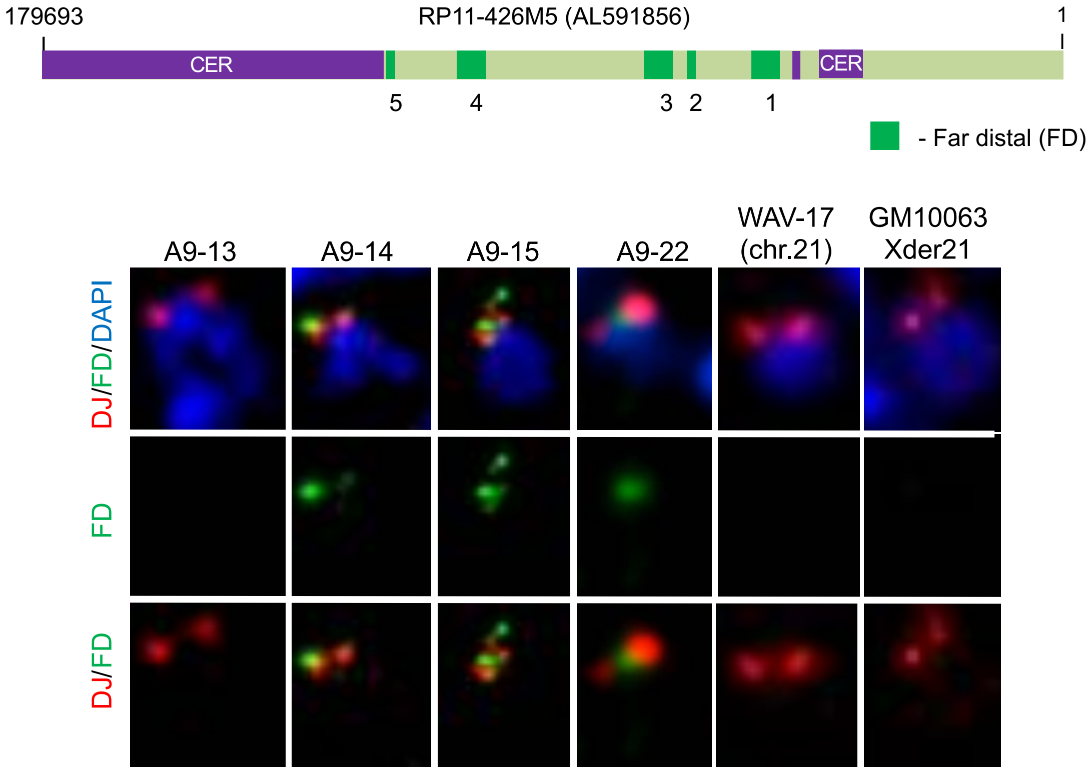

Figure S14. Confirmation of far distal sequences on human acrocentrics in the hybrid panel. Metaphase spreads prepared from mono-chromosomal hybrid lines were probed with DJ BAC CT476834 (red) and a far distal (FD) probe (green) prepared by labelling cloned PCR products (1-5) from non-repetitive regions of BAC clone AL591856. A schematic of the FD probe is shown above images of the human acrocentric chromosome present in each hybrid line. Note the absence of FD signal in A9-13, WAV17 and GM10063, and strong hybridisation signals in A9-14 and 15 as predicted by the sequence analysis (Fig. 6A). The FD hybridisation signal in A9-22 is presumably due to the second AL591856 related contig on HAS 22 in these cells (Fig. S9).

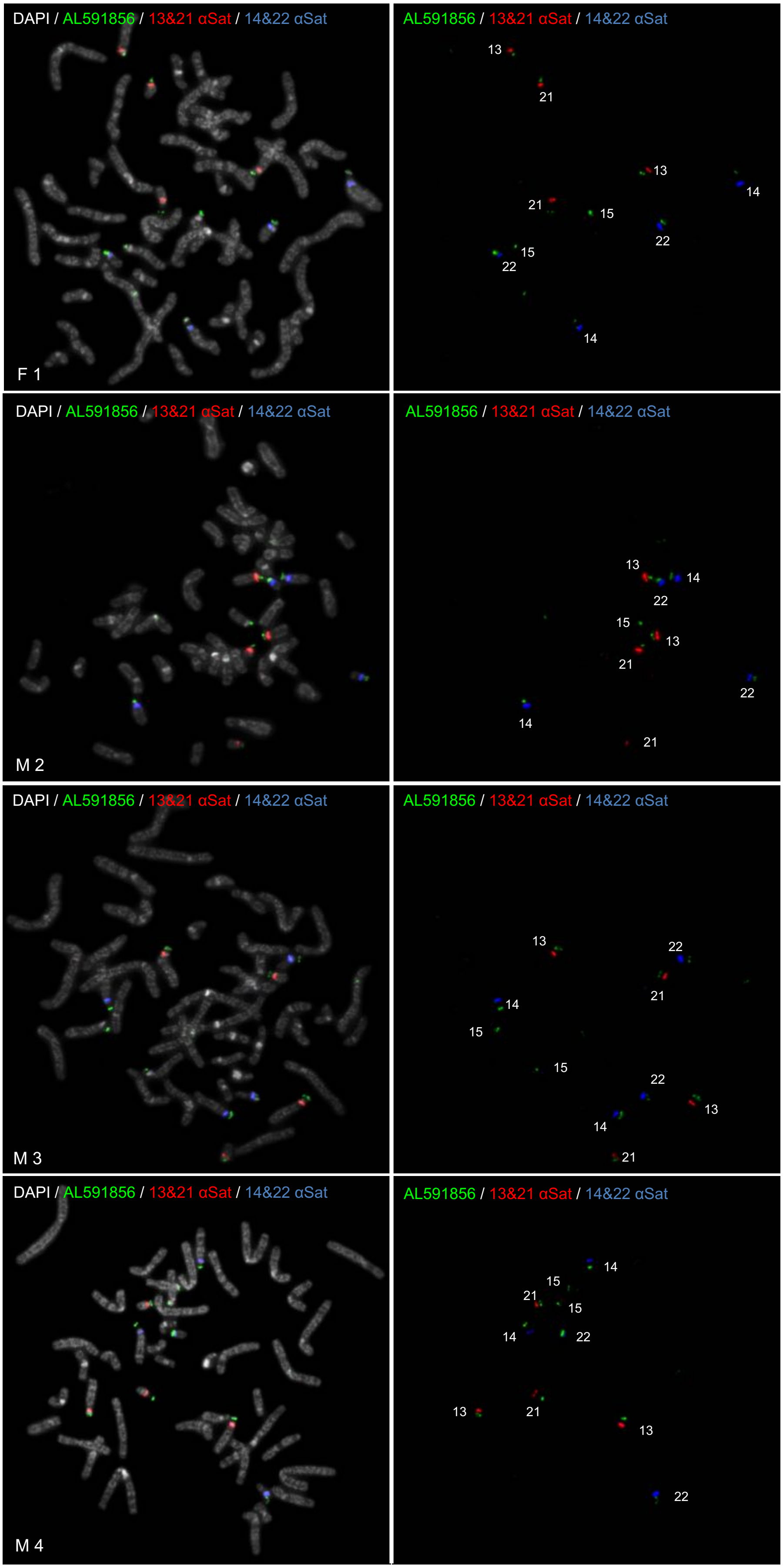

DAPI / AL591856 / 13&21 αSat / 14&22 αSat
AL591856 / 13&21 αSat / 14&22 αSat
13
21
13
14
21
15
22
15
22
14
F 1
M 2
13
14
22
15
13
21
22
14
21
M 3
13
22
21
14
15
15
22
13
14
21
M 4
14
15
21
15
14
22
13
21
13
22

Fig. S15

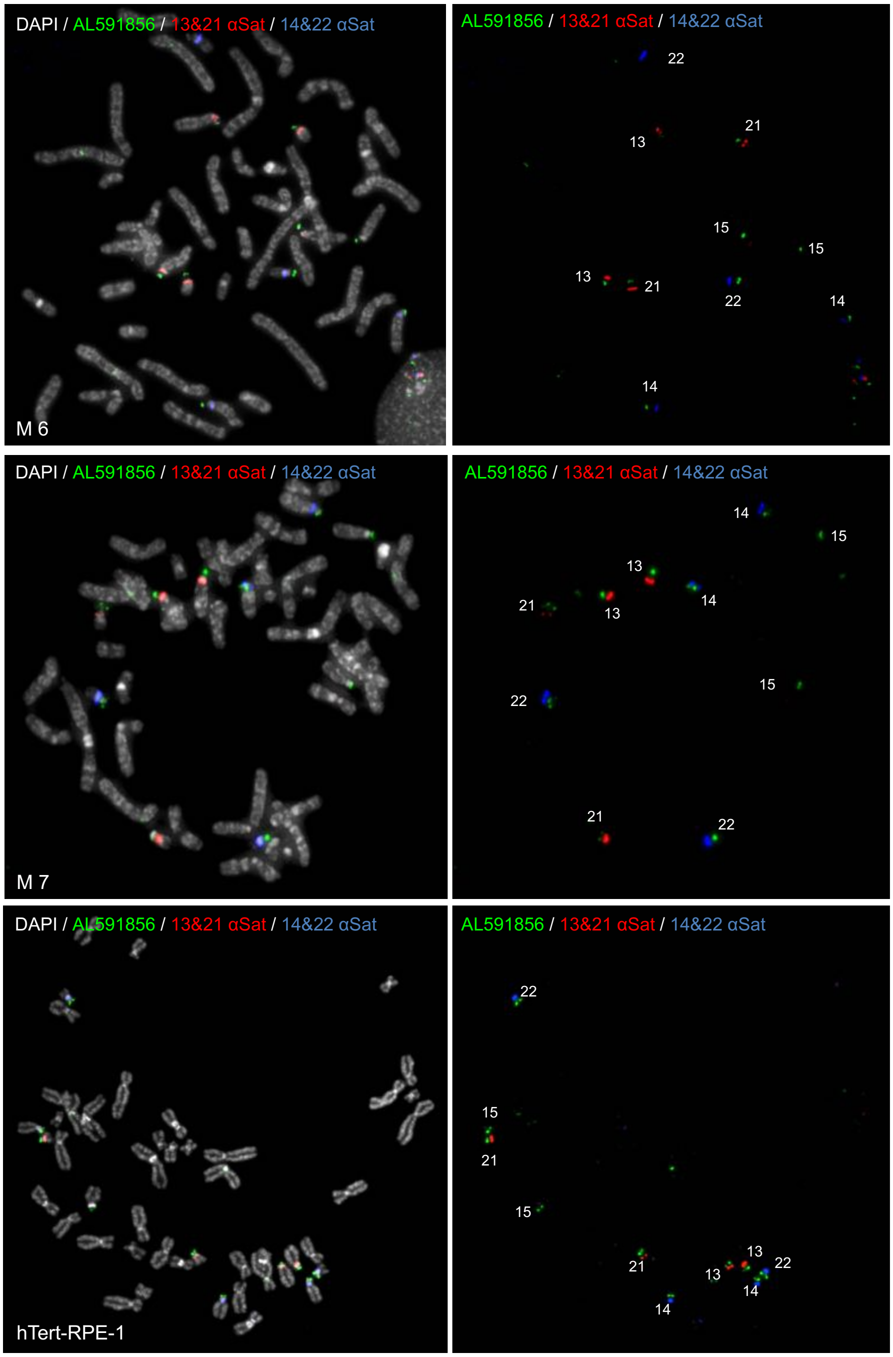

Figure S15. Variation in far distal sequences among human individuals. Normal metaphase spreads from six individual donors and from hTert-RPE1 cells were probed with BAC clone AL591856 (green) and alpha satellite probes recognising HSA13/ 21 (red) and HSA14/22 (far red, pseudo-coloured here in blue). Chromosomes were DAPI stained (pseudo-coloured in grey). Enlarged images of the 10 acrocentric p-arms from each spread are presented in Fig. 5D.

Library	Number of ROIs	Mean read length (bp)	Mean number of passes	Read bases of inserts	Mean read quality of insert
A9-13	105772	3596	9	380456833	0.9827
A9-14	114179	3167	8	361611131	0.9812
A9-14	99946	3377	8	337546560	0.9798
A9-14	103063	2757	11	284169531	0.9813
A9-14	48361	3412	8	165042683	0.9794
WAV17	43778	3469	8	151890055	0.9768
GM10063	89992	3310	10	297924023	0.9794

Table S1 Summary of sequencing statistics

Clone Name	Library	GenBank Accession	Obtained from
CH507-146P16	CHORI-507-HSA21	CT476837	
CH507-535F5	CHORI-507-HSA21	CT476834	BACPAC Resources Center
CH507-145C22	CHORI-507-HSA21	CU633906	BACPAC Resources Center
RP11-272E10	RPCI-11 Human Male	AC011841	BACPAC Resources Center
RP11-426M5	RPCI-11 Human Male	AL591856	BACPAC Resources Center
bP-2154M18	CHORI-507-HSA21	CR381535	
CH251-114B1	CHORI-251 Chimpanzee	AC194567	BACPAC Resources Center
CH251-351B7	CHORI-251 Chimpanzee	AC213064	BACPAC Resources Center
CH251-577A14	CHORI-251 Chimpanzee	AC195095	

Table S2 BAC clones used in this study

Name	Sequence	Use/location
WL1F	CACGCCTTCTCTTGCTCTCA	PCR across HERV-K in A9-22(HSA22) and WAV-17 (HSA21)
WL1r	TGCCCATGGTTTCCAGAACA	as above
GAP33F	GTCACCCCGTCTCGGAAG	PCR across GC rich gap in read coverage at ~33kb
GAP33R	TCAACAGGATCCCAAGGCAG	as above
GAP93F	GCCGAGATGGAAGGATCACC	PCR across AT rich gap in read coverage at ~39kb
GAP39R	TTCAGGCGTTCATCCACCAG	as above
GAP70F	TGGAGAAGCGAGCTTCAGTG	PCR across AT rich gap in read coverage at ~70kb
GAP70R	CTTGGGTTGTCGATGGGACT	as above
GAP202F	CATGGCCAGCATGGCAAAAA	PCR across AT rich gap in read coverage at ~202kb
GAP202R	TCTGAGGCACTGAGAGGTGA	as above
GAP223F	TGTGAGACTTTGTTGGGGGT	PCR across AT rich gap in read coverage at ~223kb
GAP223R	ATGGCCAGCATGGCAAAAAC	as above
SeqCapPB1	AATGATACGGCGACCACCGA*G*A	Pre and post capture library amplification
SeqCapPB2	CAAGCAGAAGACGGCATACG*A*G	as above * denotes phosphorothioate linkages
disnor 238f	ACAGCCCCTGTTGCCCTGCG	RT-PCR, 238.5/exon1 (disnor 238)/forward
disnor 238r	CTGCGTCTCACCAAGGCTCACC	RT-PCR, 244.3/exon3 (disnor 238/reverse
148F	GCCTCATGCTGCATTCAAGG	RT-PCR, 148/exon4 (disnor 187)/forward
148R	GTATAGAGCCGGGTTTGCGT	RT-PCR, 148/exon4 (disnor 187)/reverse
248F	GAGGAAAGGCCAGGAGGTC	RT-PCR, 248/exon3 (disnor 238)/forward
248R	TTCACCTTCCAGACACGCTC	RT-PCR, 248/exon3 (disnor 238)/reverse
AlphaSat1	GAAGCTTA(A/T)(C/G)T(C/A)ACAGAGTT(G/T)AA	Generation of chromosome specific centromere probes
AlphaSat2	GCTGCAGATC(A/C)C(A/C)AAG(A/T/C)AGTTTC	as above
ALUN1nf	TTCATCTAGGCATGGTGCTGT	PCR from AL591856 to construct FD probe 1
ALUN1nr	GTAGCTGGAGAGGTGTAGGT	as above
ALUN2nf	TCAGTTCCTTCAAGACTGCAT	PCR from AL591856 to construct FD probe 2
ALUN2nr	CCCAGAACCATCACCTGTTCT	as above
ALUN3nf	TGGGTGAGAGAGAAAGGTTTGG	PCR from AL591856 to construct FD probe 3
ALUN3nr	GCTGCAAAAGAAAGCAAAGAGC	as above
ALUN4nf	TCTGTGACAGCAACACTTAGAAC	PCR from AL591856 to construct FD probe 4
ALUN4nr	TGTGACCAATGCAGTCAGTGT	as above
ALUN5nf	GTTTTGTATGGTAGCGGAACAACT	PCR from AL591856 to construct FD probe 5
ALUN5nr	ATCCCTGGAGCATCCATCCG	as above

Table S3 Sequences of oligonucleotides used throughout this study

Sequence	Source of DNA	GenBank accession code	SRA accession code (post-capture ROIs)
HSA13 DJ	Hybrid A9-13	MG910335	SRR10403916
HSA14 DJ	Hybrid A9-14	MG910336	SRR10403915
HSA15 DJ	Hybrid A9-15	MG910337	SRR10403914
HSA21 DJ	Hybrid A9-21	MG910338	SRR10403913
HSA22 DJ	Hybrid A9-22	MG910339	SRR10403912
HSA21 DJ	Hybrid WAV17	MG910340	SRR10403910
Xder21 DJ	Hybrid GM10063	MG910341	SRR10403911
HSA14 far-distal	Hybrid A9-14	MN651987	SRR10403915
HSA15 far-distal	Hybrid A9-15	MN651988	SRR10403914
DJ Like (seq1)	*Pan troglodytes* (Vanessa)	MN646268	N/A
DJ Like (seq2)	*Pan troglodytes* (Vanessa)	MN646269	N/A
DJ Like (seq3)	*Pan troglodytes* (Vanessa)	MN646270	N/A
DJ Like (seq4)	*Pan troglodytes* (Walter)	MN646271	N/A
DJ Like (seq5)	*Pan troglodytes* (Walter)	MN646272	N/A

Table S4 Genbank accession codes and links to Sequence Read Archive (SRA)
